# Experiences and management of physician psychological symptoms during infectious disease outbreaks: a rapid review

**DOI:** 10.1186/s12888-021-03090-9

**Published:** 2021-02-10

**Authors:** Kirsten M. Fiest, Jeanna Parsons Leigh, Karla D. Krewulak, Kara M. Plotnikoff, Laryssa G. Kemp, Joshua Ng-Kamstra, Henry T. Stelfox

**Affiliations:** 1 Department of Critical Care Medicine, Cumming School of Medicine, University of Calgary & Alberta Health Services, 3134 Hospital Drive NW, Calgary, T2N4Z6 Canada; 2 Department of Community Health Sciences, Cumming School of Medicine, University of Calgary, 3134 Hospital Drive NW, Calgary, T2N4Z6 Canada; 3 O’Brien Institute for Public Health, Cumming School of Medicine, University of Calgary, 3134 Hospital Drive NW, Calgary, Alberta T2N4Z6 Canada; 4 School of Health Administration, Faculty of Health and Department of Critical Care Medicine, Faculty of Medicine, Dalhousie University, 5850 College Street, Halifax, Nova Scotia B3H4R2 Canada

**Keywords:** COVID-19, Mental health, Physicians, Review

## Abstract

**Background:**

Prior to the COVID-19 pandemic, physicians experienced unprecedented levels of burnout. The uncertainty of the ongoing COVID-19 pandemic along with increased workload and difficult medical triage decisions may lead to a further decline in physician psychological health.

**Methods:**

We searched Medline, EMBASE, and PsycINFO for primary research from database inception (Medline [1946], EMBASE [1974], PsycINFO [1806]) to November 17, 2020. Titles and abstracts were screened by one of three reviewers and full-text article screening and data abstraction were conducted independently, and in duplicate, by three reviewers.

**Results:**

From 6223 unique citations, 480 articles were reviewed in full-text, with 193 studies (of 90,499 physicians) included in the final review. Studies reported on physician psychological symptoms and management during seven infectious disease outbreaks (severe acute respiratory syndrome [SARS], three strains of Influenza A virus [H1N1, H5N1, H7N9], Ebola, Middle East respiratory syndrome [MERS], and COVID-19) in 57 countries. Psychological symptoms of anxiety (14.3–92.3%), stress (11.9–93.7%), depression (17–80.5%), post-traumatic stress disorder (13.2–75.2%) and burnout (14.7–76%) were commonly reported among physicians, regardless of infectious disease outbreak or country. Younger, female (vs. male), single (vs. married), early career physicians, and those providing direct care to infected patients were associated with worse psychological symptoms.

**Interpretation:**

Physicians should be aware that psychological symptoms of anxiety, depression, fear and distress are common, manifest differently and self-management strategies to improve psychological well-being exist. Health systems should implement short and long-term psychological supports for physicians caring for patients with COVID-19.

**Supplementary Information:**

The online version contains supplementary material available at 10.1186/s12888-021-03090-9.

## Background

Infectious disease outbreaks pose a severe threat to public health [1, 2]. A novel infectious disease, Coronavirus Disease 2019 (COVID-19), has spread rapidly globally after its emergence in late 2019, prompting The World Health Organization to declare a pandemic [3]. The transmissibility and the severity of COVID-19 make this a particularly dangerous pathogen [4], and adds to frontline healthcare professionals experiencing concern, anxiety, frustration, and fear [5–8].

A recent rapid review of 59 papers described psychological outcomes of all healthcare professionals in response to infectious disease outbreaks (e.g., COVID-19, Ebola, H1N1, H7N9, MERS and SARS) and reported that healthcare professionals caring for affected patients experienced increased levels of acute or post-traumatic stress and psychological stress [9]. This rapid review included data from eight studies on psychological outcomes of healthcare professionals from China during the COVID-19 pandemic. However, our search retuned 174 studies specific to COVID-19. This reflects the growth of the pandemic, with over 200 countries reporting COVID-19 cases and over 19 million cases worldwide. The impact of the COVID-19 pandemic has varied from country to country, depending on the public health measures enacted in each country to minimize transmission.

Prior to the COVID-19 pandemic, studies reported that physicians experienced unprecedented levels of burnout [10–15]. Burnout among physicians is reported to be as high as 70% [16], and has been reported to be higher than registered nurses (RNs) and respiratory therapists [17, 18]. The uncertainty of the ongoing COVID-19 pandemic along with the moral distress, long shifts, and a unique role with difficult triage decisions, may further exacerbate a physician’s mental well-being [19, 20]. To date, experiences and management of physician psychological symptoms during the COVID-19 outbreak have not been comprehensively described and compared between countries and with previous infectious disease outbreaks. In response to this gap in the literature, we conducted a rapid review of the experiences and management of psychological symptoms in physicians during infectious disease outbreaks to better understand and support physician health during this unprecedented time.

## Methods

We conducted a rapid review [21] to ensure timely synthesis of data in response to the COVID-19 pandemic. In the absence of a PRISMA statement for rapid reviews, we used the overall statement [22] to guide us where possible (Additional File 1) [23].

### Eligibility criteria

Eligibility criteria were defined using the “Population, Exposure, Comparison, Outcomes, Study Designs” (PECOS) components.

### Population

Physicians involved in caring for patients in any healthcare setting during an infectious disease outbreak. Due to the rapid nature of this review, other healthcare professionals were excluded.

### Exposure

Caring for patients during an infectious disease outbreak. Care could be anticipated (e.g., not having worked a shift yet) or from direct patient care experiences.

### Comparators

Any intervention, control group, or studies without a comparison group were eligible for inclusion.

### Outcomes

Any outcome that measured physician psychological symptoms (e.g., fear, anxiety, stress) or ways to manage these experiences (e.g., behaviour changes) during infectious disease outbreaks.

### Study designs

Any primary research of quantitative or qualitative design.

### Search strategy and selection criteria

The search strategies were developed by a psychiatric epidemiologist (KMF), critical care physician (HTS), and a medical librarian. We searched Medline, EMBASE, and PsycINFO from inception until November 17, 2020. The final search strategy for Medline can be found in Additional File 2. No restrictions were placed on the language or date of publication.

Titles and abstracts were imported into Endnote X9 (Clarivate Analytics), which was also used to manage full-text articles. Inclusion criteria were as follows: 1) primary research (e.g., cross-sectional studies); 2) physicians as the target population; and 3) article describes experiences or management of psychological symptoms (e.g., anxiety, fear, stress) related to infectious disease outbreaks (e.g., COVID-19, SARS [Severe Acute Respiratory Disease Syndrome], H1N1). Exclusion criteria included: 1) studies of HIV or AIDS; and 2) physician perceptions or attitudes towards vaccination. Titles and abstracts were reviewed by one of three reviewers (KK, KP, LK) [24]. All abstracts selected by any reviewer moved onto the full-text stage. Full-text review was conducted independently and in duplicate. Disagreements (e.g., include/exclude article or reason for exclusion) were resolved by discussion or involvement of another reviewer as necessary.

### Data abstraction

We abstracted data on publication (e.g., year of publication), study (e.g., location of data collection, setting, time of data collection), and participant characteristics (e.g., age, sex, specialty). Outcome data on experiences or management of physician psychological symptoms were also abstracted. The data abstraction form was developed and piloted by the study team. The data abstraction form, including quality assessment, was piloted on 10% of the included studies to ensure agreement. All data were abstracted independently and in duplicate. Any discrepancies between independently abstracted data were resolved by discussion or involvement of another reviewer as necessary. Data are reported using descriptive statistics.

### Study quality appraisal

The quality of included quantitative studies was assessed using the Newcastle-Ottawa Scale, including the extension for cross-sectional studies [25, 26]. This is an eight- (cohort studies) or seven-item (cross-sectional studies) checklist. Studies were rated from 0 to 10 with a higher number indicating lower risk of bias and better study quality (very good: 9–10 points; good: 7–8 points; satisfactory: 5–6 points; unsatisfactory: 0 to 4 points). The Joanna Briggs Institute Checklist for Qualitative Research was used to determine the quality of qualitative studies [27].

## Results

### Literature search

**Fig. 1:**
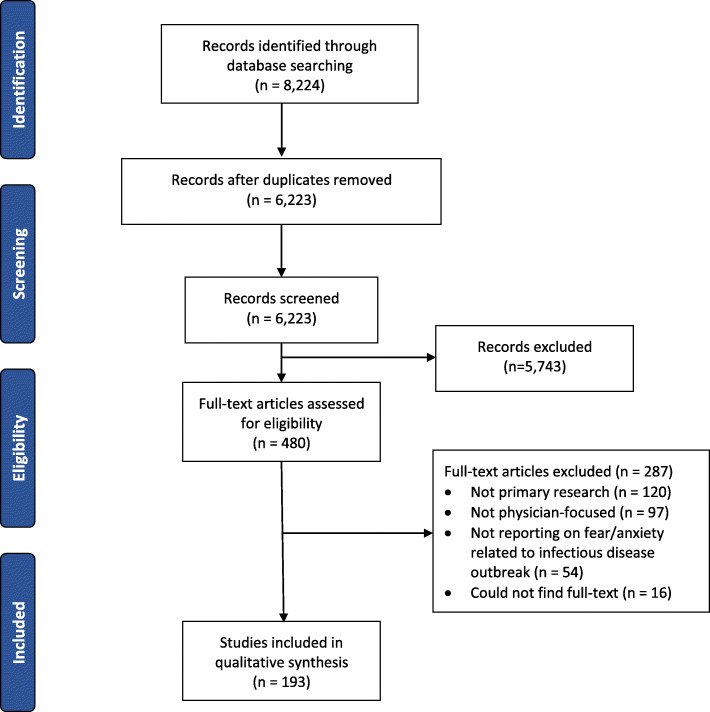
PRISMA diagram of study selection

Following the removal of duplicates, the search yielded 6223 citations (Fig. 1). Of these, 480 articles were reviewed in full-text. The most common reasons for exclusion (*n* = 287) at the full-text stage were that the research was not primary research (*n* = 120), was not focused on physicians (*n* = 97), or not reporting on psychological symptoms associated with an infectious disease outbreak (*n* = 54). A total of 193 papers were included in the rapid review (Additional File 3) [28–89]. Most studies had a cross-sectional design (*n* = 181, 93.8%). All other study designs are listed in Additional File 3.

### Publication, study, and participant characteristics

**Fig. 2:**
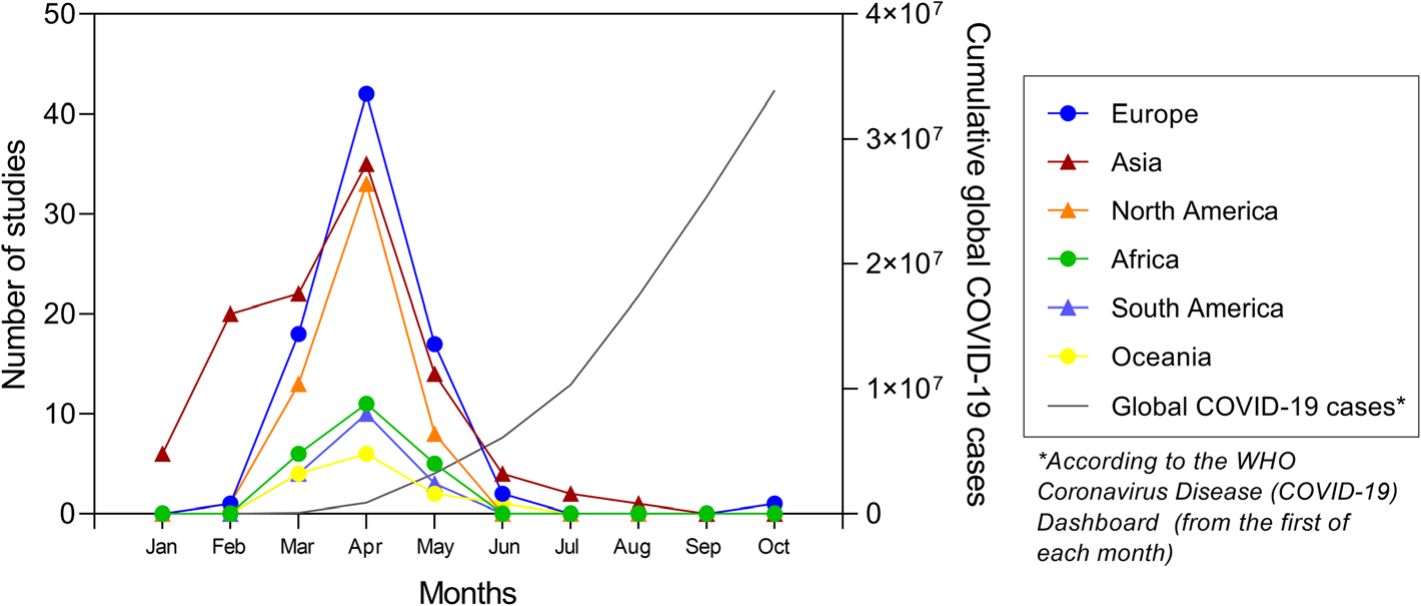
Number of cumulative studies from different continents reporting on the psychological symptoms in physicians during the COVID-19 pandemic compared to the number of active global COVID-19 cases in 2020. Nineteen studies did not report a date range for data collection

Of the 193 included studies (of 90,499 physicians), the majority focused on COVID-19 (*n* = 172, published in 2020) or SARS (*n* = 13, published between 2004 and 2009), followed by Influenza A virus (*n* = 5; *n* = 2 H1N1 [swine influenza], 2010–2011; *n* = 2 H5N1 [avian influenza], 2008; *n* = 1 H7N9 [avian influenza], 2017), Ebola (*n* = 2, published between 2016 and 2017), and Middle East Respiratory Syndrome (MERS) (*n* = 1, 2019). Most included studies were conducted in Asia (*n* = 94) or North America (*n* = 30) [one study was conducted in both], Europe (*n* = 36), Africa (*n* = 8), South America (*n* = 4), Oceania (*n* = 3), collectively representing 57 different countries. Studies that focused on COVID-19 were conducted in 55 different countries from six different continents (Africa, Asia, Europe, North America, Oceania, South America) throughout the COVID-19 pandemic (Fig. 2) and included 87,667 physicians. Studies included physicians from a variety of settings and specialties (e.g., general practitioners, ICU, emergency room, urologists, oncologists, opthamologists, surgeons, etc.) (Additional File 3). Seventy studies reported on trainee physician (i.e., resident, fellow) experiences with psychological symptoms during infectious disease outbreaks.

### Experiences with physician psychological symptoms during infectious disease outbreaks

All studies (*n* = 193) reported on physician experiences with psychological symptoms during infectious disease outbreaks, with consistent experiences reported across different disease outbreaks. The most common method to identify psychological symptoms (e.g., anxiety, depression, stress, isolation, worry, fear, stigma) was a validated questionnaire (*n* = 120) or survey (*n* = 91). The most common psychological symptoms measured with surveys or validated questionnaires reported included anxiety (*n* = 103), depression (*n* = 73), stress (*n* = 68), fear (*n* = 61), burnout (*n* = 25), mental distress/health (*n* = 21), and post-traumatic stress disorder (PTSD) (*n* = 14) (Additional File 3). Most COVID-19 studies reported on physicians from USA (*n* = 23) [41, 52, 66, 70, 77, 90–107], China (*n* = 22) [15, 49, 54–56, 65, 68, 73, 86, 89, 102, 108–118] followed by India (*n* = 21) [40, 50, 58, 63, 67, 71, 102, 119–132] [41, 52, 66, 70, 77], Italy (*n* = 10) [32, 94, 102, 125, 133–138], and Turkey (*n* = 11) [35, 44, 94, 139–146] [28, 30, 32, 34, 36, 37, 42, 45, 47, 48, 59, 60, 62, 80, 87]. .Sixteen studies reported on physicians from multiple countries [47, 51, 69, 94, 102, 106, 125, 133, 147–154].

**Fig. 3:**
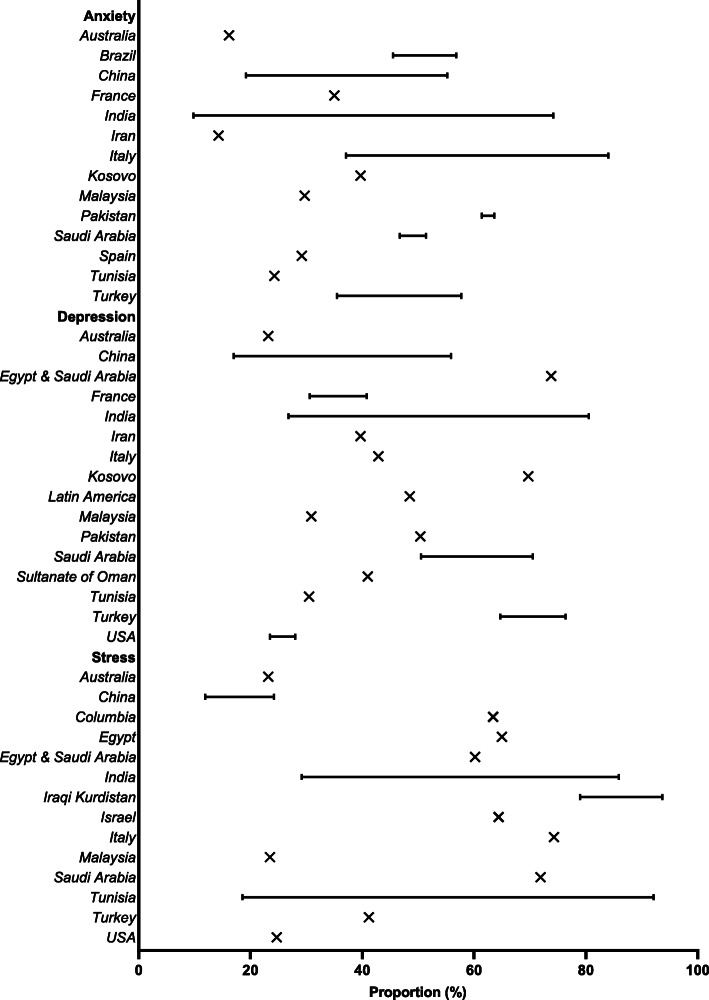
Proportions of physicians experiencing COVID-19 related psychological symptoms (i.e., any symptoms of anxiety, depression, or stress measured on a validated scale [i.e., mild, moderate, and severe]) grouped by country

Ninety-seven (97/172, 56.4%) studies from 43 countries (most from China [*n* = 17], USA, [*n* = 11], Turkey [*n* = 9], India [*n* = 8], Pakistan [*n* = 7], or Italy [*n* = 6]) reported physician anxiety during the COVID-19 pandemic. Most studies used the Generalized Anxiety Disorder (GAD-2/GAD-7)(*n* = 33), survey/interview (*n* = 26, e.g., “Do you feel anxious when you case for COVID-19 patients” [35] or “Rate your level of anxiety regarding the COVID-19 outbreak on a scale of 1 to 10 [91]), Depression, Anxiety and Stress Scale-21 Items (DASS-21)(*n* = 9), Hospital Anxiety and Depression Scale (HADS)(*n* = 9), State Trait Anxiety Inventory (STAI) (each *n* = 4), along with other measurements (Additional File 3). The proportion of physicians who experienced symptoms of anxiety (i.e., any symptoms of anxiety measured on a validated scale [i.e., mild, moderate, and severe]) was reported in 34 studies, and ranged from 14.3% (Iran) to 92.3% (Saudi Arabia and Egypt) (Fig. 3), with many individuals (9.8% [India] to 39.3% [Colombia]) having clinically significant symptoms (i.e., described as having clinically significant symptoms in the study or meeting the cut-off for clinically significant symptoms for the specific scale used [e.g., GAD-7 score ≥ 10]). During other infectious disease outbreaks, the proportion of physicians with self-reported symptoms of anxiety related to the infectious disease outbreak ranged from 29.1% (SARS) to 67.0% (Ebola).

Seventy-two studies (72/172, 41.9%) from 37 countries (most from China [*n* = 12], India [*n* = 8], USA [*n* = 6], Turkey [*n* = 5],) reported physician depression related to the COVID-19 pandemic. Most studies used the Patient Health Questionnaire-9 (PHQ-2/PHQ-4/PHQ-9)(*n* = 31), DASS-21 (*n* = 10), survey (*n* = 9, e.g.,“I feel depressed” [5-point Likert scale]), HADS (*n* = 9), along with other measurements (Additional File 3) [155]. The proportion of physicians who experienced symptoms of depression (i.e., any symptoms of depression measured on a validated scale [i.e., mild, moderate, and severe]) was reported in 31 studies, and ranged from 17% (China) to 80.5% (India) (Fig. 3), with many individuals (7.2% [China] to 62% [Turkey]) having clinically significant symptoms (i.e., described as having clinically significant symptoms in the study or meeting the cut-off for clinically significant symptoms for the specific scale used [e.g., PHQ-9 score ≥ 10]). The proportion of physicians with self-reported symptoms of depression was not reported for other infectious disease outbreaks included in this review.

Sixty-four studies (64/172, 37.2%) from 33 countries (most from India [*n* = 13], USA [*n* = 9]) reported on physician stress (Fig. 3) related to the COVID-19 pandemic. Most studies used a survey (*n* = 29, e.g., “My daily life has become more stressful due to the COVID-19 pandemic” [156]), Patient Stress Scale (PSS-10, *n* = 17), DASS-21 (*n* = 9), along with other measurements (Additional File 3). The proportion of physicians who experienced symptoms of stress (i.e., any symptoms of stress measured on a validated scale [i.e., mild, moderate, and severe]) was reported in 26 studies, and ranged from 11.9% (China) to 93.7% (Iraqi Kurdistan) (Fig. 3). Symptoms of stress among physicians during other infectious disease outbreaks ranged from 31.8% (SARS) to 100% (Ebola).

Twenty-four studies from 12 countries reported on the proportion of physicians with symptoms of burnout related to the COVID-19 pandemic, which ranged from 14.7% (Canada) to 76% (Romania). Nine studies from seven countries reported on the proportion of physicians with symptoms of PTSD related to the COVID-19 pandemic, which ranged from 24.3% (China) to 75.2% (Turkey). Symptoms of PTSD and burnout were reported for other infectious disease outbreaks 13.2% (SARS) and 36.4% (H1N1), respectively.

Sixty-one studies reported on fear related to an infectious disease outbreak [29–31, 35–37, 43, 46, 53, 55, 61, 64, 72, 74, 78–83, 85–87, 94, 95, 99, 100, 107–109, 115, 117, 119, 123, 128, 131, 132, 134, 142, 146, 147, 155–173]. Most of the studies (*n* = 52) reported physician’s fear of infecting themselves or their family. The greatest sources of distress for emergency department physicians during SARS were the spread of the virus and the health of their family [83] and during the MERS outbreak, many Saudi Arabian physicians reported a great source of their anxiety was worry over their family acquiring the illness [31]. More than one-quarter (27.5%) of academic physicians in Canada were concerned about SARS spreading to their family [46], while 89% of Egyptian physicians were concerned their family would contract H1N1 from them [43]. In Hong Kong, female physicians were more worried about infecting their families with SARS than male physicians [82]. Two months following the SARS outbreak, physicians reported that their greatest concern were fears of infecting self, family, and other loved ones [81]. Saudi Arabian physicians with past pandemic experience were less likely to experience fear during the COVID-19 pandemic [30].

### Factors associated with psychological symptoms during infectious disease outbreaks

One hundred studies reported on factors associated with psychological symptoms during infectious disease outbreaks. Female sex (compared to male) was the factor most commonly associated with worse psychological symptoms in physicians [30, 41, 44, 48, 63, 66, 71, 77, 89, 98, 112, 122, 127, 128, 138, 142, 144, 148, 163, 165, 170, 174–189]. Other factors associated with poorer psychological outcomes included direct patient contact [28, 32, 34, 44, 46, 53, 59, 64, 71, 81, 95, 98, 112, 122, 130, 163, 169, 175, 182, 183, 186, 190–192] [30, 34, 41, 44, 48, 59, 63, 66, 71, 77, 89], single marital status (compared to married) [39, 44, 59, 63, 98, 122], younger age [33, 44, 47, 48, 50, 67, 77, 81, 82, 136, 148, 170, 174, 177, 179, 181, 191, 193–196], and more junior career stage (e.g., residents compared to staff) [28, 41, 43, 44, 48, 65, 77, 100, 120, 122, 128, 129, 133, 136, 170, 174, 181, 185, 196, 197]. During COVID-19, feelings that there was inadequate PPE supply at their hospital was associated with worse psychological outcomes [107, 143, 147, 157, 163, 165, 170, 178, 187, 188]. Physicians working more days, with higher patient volumes, or with increased duties than before than pandemic also had an increased prevalence of psychological symptoms [96, 101, 122, 128, 149, 187, 192, 195, 198]. Eighteen studies reported on insomnia [32, 35, 36, 54, 65, 69, 88, 114, 128, 145, 149, 163, 175, 177, 181, 188, 192, 194], which is associated with psychiatric disorders and is a risk factor for depression and anxiety [15, 32, 54, 65, 69, 199, 200]. During the SARS outbreak, physicians of Asian descent felt more stigmatized than their Caucasian colleagues (Canada) [15, 32, 35, 36, 46, 54, 65, 69, 88, 114, 128, 145, 149, 163, 175, 177, 181, 188, 192, 194, 199, 200].

Eleven studies [32, 54, 74, 81, 82, 84, 85, 94, 136, 143, 201] focused on the experiences of family physicians during infectious disease outbreaks. Family physicians practicing in Singapore during the H5N1 outbreak feared there would be inadequate staff to manage patient demand [85]. During the SARS outbreak, the majority of surveyed family physicians in both Hong Kong and Canada reported having no infection control training (80%) and lacked confidence in dealing with SARS (~ 70%). Many concerns centered around training, availability, and use of personal protective equipment (PPE). Family physicians felt they did not have sufficient training or experience with appropriate use of PPE, which they felt limited its effectiveness [74]. The shortage of PPE heightened their insecurities and some physicians recycled PPE or wore PPE for prolonged periods of time, which caused physical discomfort [74, 143]. Some family physicians reported not following protective procedures, such as hand washing or wearing gowns [82].

### Management of physician psychological symptoms during infectious disease outbreaks

Nearly one-third (*n* = 64, 33.2%) of the studies evaluated ways physicians managed psychological symptoms during infectious disease outbreaks. Groups of physicians from several countries (China [108], India [40], Saudi Arabia [31], Egypt [43], Singapore [39, 74, 85], and Hong Kong [64]) reported voluntarily engaging in practices such as increased hand washing and wearing of PPE, even if it was not yet required by their workplaces (*n* = 8). Recognition of service from the government was also indicated by physicians in China [108] and Singapore [53] as a positive buffer to experiencing negative psychological symptoms. Avoiding outbreak related news and social media was also reported by physicians in China [56, 108] and Saudi Arabia [30] (*n*=3) conducted during the COVID-19 pandemic.

Physicians reported the importance of having social support from friends, family, colleagues, and professionals (e.g., counsellors) in 29 studies [28, 30, 39, 43, 44, 48, 56, 58, 67, 72, 78, 83, 91, 99, 102, 106, 115, 119, 131, 150, 155, 163, 172, 192, 202–205]. Several studies (*n* = 29) described positive personal coping strategies such as maintaining a positive attitude and resilience [37, 46, 83, 100, 106, 108, 136, 202, 205, 206], practicing self-care (e.g., physical activity, eating well, resting, engaging in activities they enjoy) [30, 35, 56, 65, 70, 91, 102, 103, 117, 119, 129, 131, 150, 151, 207], and engaging with religious practices [39, 46, 131, 205] as a way to mitigate negative psychological outcomes. In contrast, two studies reported that physicians engaged in avoidance coping strategies (e.g., screaming, crying, denial, self-blame, disengaging, substance abuse, etc.) [83, 106, 108, 127, 202, 203, 205].

Specific to COVID-19, physicians in China [56], France [196], India [40, 50, 58], Pakistan [157, 192], South Korea [184], Tunisia [192], Turkey [44], and USA [99, 103, 105, 203] expressed desire for their organizations to provide access to psychological support through counselling programs or support hotlines (*n* = 13). Communication and appreciation from hospital administrators (*n* = 4) was indicated as an important gesture to help foster feelings of gratitude for physicians in China [108, 115], Colombia [201], and the USA [52] [52, 58, 62]. Physicians in China [108], France [28, 188], India [129], Israel [208], Pakistan [182], Saudi Arabia [30], Turkey [44], and the USA [52] indicated the importance of supportive workplace environments that provide an adequate supply of PPE, proper training, and comprehensive communication about infection prevention strategies.

### Study quality assessment

Of 181 cross-sectional studies, scores ranged from 1 (*n* = 1, 0.6%) [86] to 9 (*n* = 4, 2.2%) [128, 140, 188, 201] with a median score of 5 (Additional File 4). Twenty-eight studies justified their sample size [54, 59, 70]. The majority of studies did not compare between respondents and non-respondents (*n* = 159, 87.8%) or include 95% confidence intervals (*n* = 100, 55.2%). The five included cohort studies [55, 57] had scores ranging from 4 (*n* = 1, 20%) to 6 (*n* = 4, 80%. Only one described the ascertainment of exposure [55]. The six included qualitative studies [56, 72, 74, 115, 204, 209] were considered to be of sufficient methodological quality. One study had a pre-test, post-test study design and was considered to be of sufficient methodological quality [112].

## Discussion

In this rapid review, we summarized the literature from 193 studies on physician experiences and management of psychological symptoms during infectious disease outbreaks. Results were consistent, with over 90,000 physicians surveyed during and after seven different infectious disease outbreaks (SARS, H7N9, H5N1, H1N1, MERS, Ebola, COVID-19) in 57 countries over the last 17 years. The burden of psychological symptoms in physicians was high, with anxiety symptoms affecting as many as 92.3% [210] of physicians and symptoms of PTSD in as many as 75.2% [145]. Female, younger, more junior physicians were reported to be at higher risk of more symptoms and/or more severe symptoms, as were those physicians who were in direct contact with infected patients. Though, these associations should be interpreted with caution as future prospective studies are needed to confirm the associations between demographic variables/career stage of physicians and adverse psychological symptoms. Worry about their families being infected during the outbreak was the greatest source of physician psychological symptoms. Concerns about the availability and use of PPE predominated the distress of family physicians. How physicians managed psychological symptoms was highly variable and included tangible acts such as self-isolating away from family, following protocols, and receiving emotional support from family members. Some experts estimate that the pandemic could continue for much longer- at least 24 months [211]. Given the magnitude and long-term projection of the COVID-19 pandemic, and the number of physicians involved, the impact on performance, burnout, quality of life, and personal relationships could be large.

COVID-19 is affecting physicians through infection, psychological symptoms, and an interplay between the two. COVID-19 appears to affect older individuals disproportionately [212–214] and biological sex is associated with higher COVID-19 disease severity and death rates in males [213, 215]. The physician workforce may be disproportionately affected by COVID-19; 20% of healthcare workers in Italy [216] had COVID-19 and China’s National Health Commission reports that over 3300 healthcare workers have been infected as of early March [217]. To some extent this describes the healthcare workforce of many specialities directly caring for patients with COVID-19 in hospital, including critical care, emergency, and infectious disease medicine [218–220]. In contrast, family medicine, which may be the first point of medical contact for many patients, is a specialty with a greater proportion of female physicians [221]. The results of this rapid review suggest that who manifests psychological symptoms during this infectious disease outbreak may differ by age and specialty. Younger, more junior physicians (i.e., residents) are at greater risk of developing psychological symptoms during infectious disease outbreaks. Residency training programs should ensure psychological support for trainees caring for patients during COVID-19.

Further to the above recommendation, this review offers important learnings that individual physicians and health systems can use to immediately inform their understanding and management of physicians’ psychological symptoms during an infectious disease outbreak. First, physicians should take up and apply the knowledge that psychological symptoms are common across the medical profession, manifest in various ways (e.g., anxiety vs. fear) and have specific triggers (e.g., close work with infected patients) in their day-to-day lives. For instance, this conceptualization should be used to help normalize experiences of psychological symptoms in oneself, enable recognition of symptoms in colleagues, and catalyze open dialogue around mental health and wellbeing across physician groups. Second, health systems must immediately prioritize the development of short and long-term supports for individual physicians, both in terms of recognition (e.g., education of psychological signs and symptoms) and treatment (e.g., online supports) options [222]. While some health systems have already begun to prioritize this type of programming [223, 224], there is much more that can be done to further these efforts [225]. As the impact is differential based off of area of practice (i.e., direct contact with infected patients) systems should focus their efforts on those physicians at the front lines of the pandemic. Finally, it is reasonable to extrapolate from this review that health systems should implement a long-term planning process to tackle some of the larger issues identified as triggers of physician psychological symptoms during an infectious disease outbreak, including options for appropriate accommodation away from family members when treating high-risk patients, and accessible training and access to PPE for all family physicians.

A major strength of our rapid review is the timely synthesis of evidence for physicians, health systems, and policy makers on physician psychological symptoms during the COVID-19 pandemic. This rapid review followed established standards [21], which included a comprehensive literature search in multiple databases and full-text review, data extraction, and quality assessments performed independently, and in duplicate. No restrictions were placed on language of publication, and we were able to capture literature from many countries affected by recent infectious disease outbreaks (e.g., China, Singapore, Hong Kong, United States, Canada). While the review by Kisely and colleagues [9] previously summarized psychological effects on healthcare workers overall, our review reports on more recent publications and compares the outcomes of the COVID-19 pandemic with previous infectious disease outbreaks. Our review also focuses on physicians, who may be uniquely impacted during infectious disease outbreaks as they are required to take on a high level of responsibility with patient care. As with all rapid reviews there are limitations to consider. It is possible some studies were missed in the search, though results were consistent in all included studies, regardless of the country or infectious disease outbreak. We restricted our search to include selected infectious disease outbreaks and, as such, the results may not be generalizable to other outbreaks (e.g., HIV/AIDS). Due to the rapid nature of this review, we restricted our search to physicians and therefore the results may not be generalizable to other healthcare workers, such as nurses or allied health professionals. The strategies described to manage physician psychological symptoms are anecdotal (i.e., self-reported strategies) and further studies are needed to test their effectiveness. The evidence was predominately the result of cross-sectional surveys, though in the rapidly changing context of infectious disease outbreaks this is likely the most feasible study design to employ.

## Conclusion

This rapid review demonstrates that the burden of psychological symptoms in physicians during an infectious disease outbreak is high; half of physicians experience anxiety and one in five experience symptoms of PTSD. Physicians should be aware that psychological symptoms during an infectious disease outbreak are common, manifest in different ways, and have specific triggers. Health systems must prioritize psychological supports for physicians during and after infectious disease outbreaks and outside of outbreaks, plan to tackle the issues that place physicians at greatest risk.

## Supplementary Information

**Additional File 1.** PRISMA Checklist.

**Additional File 2.** Medline Search.

**Additional File 3 **Study characteristics (*n* = 193).

**Additional File 4 **Quality analysis of included studies (*n* = 193).

## Data Availability

The datasets used and/or analysed during the current study are available from the corresponding author on reasonable request.
