# Supplementary material for: Experiences and management of physician psychological symptoms during infectious disease outbreaks: a rapid review
Source: BMC Psychiatry. 2021 Feb 10;21:91. doi: 10.1186/s12888-021-03090-9 (PMC7875435; doi:10.1186/s12888-021-03090-9)
Supplement: Supplementary file 2 — Additional File 2. Medline Search. [file 12888_2021_3090_MOESM2_ESM.pdf]

Additional File 2. Medline Search

1. exp physician/ or physician*.mp. or "medical trainee".mp. or "medical trainees".mp. or doctor*.mp. or consultant*.mp. or resident*.mp. or fellow*.mp. or registrar*.mp. or "general practitioner".mp. or "general practitioners".mp.
2. stress disorders, traumatic/ or stress disorders, post-traumatic/ or stress disorders, traumatic, acute/
3. exp Stress, Psychological/
4. Stress, Physiological/
5. Adaptation, Psychological/
6. fear/ or panic/
7. anxiety/ or catastrophization/
8. exp Anxiety Disorders/
9. Mental Health/
10. "Trauma and Stressor Related Disorders"/
11. (stress* or anxiet* or fear* or panic* or nervous* or dread* or apprehensi* * or pressure* or overwhelm*).mp.
12. PTSD.mp.
13. catastroph*.mp.
14. (mental adj2 (health or ill or illness)).mp.
15. or/2-14
16. exp Disease Outbreaks/
17. Hemorrhagic Fever, Ebola/
18. influenza a virus, h1n1 subtype/ or influenza a virus, h3n2 subtype/
19. Influenza, Human/
20. Influenza A virus/
21. Orthomyxoviridae/
22. SARS Virus/
23. Severe Acute Respiratory Syndrome/
24. exp Coronavirus/
25. Coronavirus Infections/
26. Middle East Respiratory Syndrome Coronavirus/
27. Coronaviridae/
28. (pandemic* or epidemic* or outbreak* or out break* or (out adj2 break*) or ebola* or h1n1 or h3n2 or influenza A or influenza virus or SARS or severe acute respiratory syndrome or MERS or middle east respiratory syndrome or coronavirus* or wuhan or beijing or shanghai or 2019-nCoV or nCov or COVID-19 or SARS-CoV-2).mp.
29. (pneumonia.mp. or exp pneumonia/) and Wuhan.mp.
30. or/16-29
31. 1 and 15 and 30
