# Supplementary material for: Experiences and management of physician psychological symptoms during infectious disease outbreaks: a rapid review
Source: BMC Psychiatry. 2021 Feb 10;21:91. doi: 10.1186/s12888-021-03090-9 (PMC7875435; doi:10.1186/s12888-021-03090-9)
Supplement: Supplementary file 3 — Additional File 3 Study characteristics (n = 193). [file 12888_2021_3090_MOESM3_ESM.pdf]

Additional File 3. Study characteristics (n=193)

Author (year)	Study design	Continent (Country)	Outbreak	Physician characteristics				Measurement scales used	Main outcome(s)
				Sample Size	Type(s) of physician	Amount of work experience (years)	Proportion female / male		
**COVID-19**									
**Enyama (2020)(1)**	Cross-sectional survey	Africa (Cameroon)	COVID-19	118	Pediatricians	Mean yrs of practice: 11.9 +/- 8.7 yrs	61.2/38.6	Survey	More than half (65.3%) of the pediatricians interviewed were ''very afraid'' or ''extremely afraid'' of being infected with SARS-Cov-2, respectively 45.5% and 19.8%. The most frequent reasons included fear of infecting their relatives (85.1%) and of developing a severe form of the disease (43.6%).
**Ali (2020)(2)**	Cross-sectional survey	Africa (Egypt)	COVID-19	193	Frontline, Anesthesia, ICU	NR	78.2/19.8/2 (Unspecified)	PSS-10[4]	65% of participants experienced high levels of psychological distress during this pandemic. The median PSS-10 score was 21 in the registrar group and 18 in the consultant group. The number one stressor amongst participants was the fear that they would get sick and then pass COVID-

									19 on to their family and friends. This fear was exacerbated by the sense that there was little protocol or direction on how to handle suspected or confirmed cases of COVID-19.
**Bakry (2020)(3)**	Cross-sectional survey	Africa (Egypt)	COVID-19	77	Cardiothoracic surgeons	NR	2.6/97.4	Survey	Most physicians had concerns about infecting family and other patients by carrying the virus. They reported high rates of using PPE to prevent infection.
**Elkholy (2020)(4)**	Cross-sectional survey	Africa (Egypt)	COVID-19	502 HCP	60.0% were physicians<br><br>Among physicians:<br>126 (41.9%) were working as chest specialists<br>38 (12.6%) were ICU specialists<br>87 (28.9%) were internal medicine/pediatric specialists<br>40 (13.3%) were laboratory/radiology specialists<br>10 (3.3%) were surgery specialists	NR	50/50	PHQ[1], GAD-7[2], ISI[3], PSS[4]	Many HCW had symptoms of anxiety, insomnia, depression, and stress. Women were at higher risk compared to men. Women experienced symptoms of severe anxiety (odds ratio [OR], 1.85; 95% CI, 1.12–3.05; p=.016), severe depression (OR, 2.013; 95% CI, 1.17–3.4; p=.011), and severe stress (OR, 2.68; 95% CI, 1.5–4.6; p<.001). Of all the hospitals surveyed, fever

									hospital workers were also more likely to suffer from severe depression (OR, 1.52; 95% CI, 1.11–2.09; p<.01), compared to quarantine hospital workers.
**Khattab & A. (2020)(5)**	Cross-sectional survey	Africa (Egypt)	COVID-19	90	Spine Surgeons	Residents: 4 (4.4%)<br>Fellows: 16 (17.8%)<br>Consultants: 70 (77.8%)	0/100	Survey	Egyptian spine surgeons were negative affected by the COVID-19 global pandemic. Nearly one-quarter of spine surgeons stopped the outpatient clinic due to fear of being infected by COVID-19
**Elhadi (2020)(6)**	Cross-sectional survey	Africa (Libya)	COVID-19	309	Surgical specialty:<br>General & GI Surgery, N = 74 (23.9%)<br>Trauma & Emergency n= 100 (32.4%)<br>Orthopedic n= 40 (12.9%)<br>Urology n=10 (3.2%)<br>Head & Neck n= 9 (2.9%)<br>Cardiothoracic n= 6 (1.9%)<br>Plastic Surgery n= 4 (1.3%)<br>Neurosurgery n= 6 (1.9%)<br>Other n = 60 (19.4%)	< 5 yrs = 201 (65%)<br>≥ 5 yrs = 108 (35%)	31.4/68.6	PHQ-9[1], GAD-7[2]	Symptoms of anxiety and depression were prevalent among surgical staff during COVID-19.

**Fekih-Romdhane (2020)(7)**	Cross-sectional survey	Africa (Tunisia)	COVID-19	210	Medical Residents	NR	70.5/29.5	DASS-21[5], IES-R[6], ISI[3], MSPSS[7]	The DASS scores revealed that 30.5%, 24,3% and 18.6% of the participants reported severe or extremely severe levels of depression, anxiety and stress, respectively. Moreover, 18.6% of participants had scores more than 33 (threshold cut-off) on IES-R scale, indicating a probable diagnosis of PTSD. The ISI scores revealed that 41.4% of residents had symptoms of insomnia
**Halayem (2020)(8)**	Cross-sectional survey	Africa (Tunisia)	COVID-19	191	Frontline	0.5-37 years	80.9/19.1	PSS-10[4]	A higher stress score was correlated with females and physicians in training. Physicians who trusted the national police management of the COVID-19 outbreak were significantly less stressed.
**Arafa (2021)(9)**	Cross-sectional survey	Africa/Asia (Egypt, Saudi Arabia)	COVID-19	206	Frontline	NR	NR for physicians only	DASS-21[5]	Physicians reported experienced anxiety, depression and stress, as well as inadequate sleep.

**Cai (2020)(10)**	Cross-sectional survey	Asia (China)	COVID-19	233	Frontline	NR	NR	Likert scale questions	During the COVID-19 pandemic, doctors were less nervous or frightened of the ward compared to nurses.
**Huang (2020)(11)**	Cross-sectional survey	Asia (China)	COVID-19	70	Frontline	NR	NR	SAS[8], PTSD-SS[9]	High incidence of physician anxiety and stress disorder.
**Juan (2020)(12)**	Cross sectional survey	Asia (China)	COVID-19	195	Hospital physician	NR	NR for physicians only	IES-R[6], GAD-7[2], PHQ-9[1], Y-BOCS[10], PHQ-15[1], Factor test designed according to DSM IV criteria	Hospital physicians in China experienced symptoms of anxiety, depression and psychological distress during the COVID-19 pandemic. There was no difference in the median scores between physicians and nurses.
**Lai (2020)(13)**	Cross-sectional survey	Asia (China)	COVID-19	493	Frontline	Level of experience: Junior: n = 153 (31.0%) Intermediate: n = 187 (37.9%) Senior: n = 180 (14.3%)	54.8/45.2	PHQ-9[1], GAD-7[2], ISI[3], IES-R[6]	Physicians had lower scores for psychological symptoms compared to nurses sampled from the same hospitals.
**Li (2020)(14)**	Cohort	Asia (China)	COVID-19	385	Residents	Range: 0-6 months	64.0/36.0	PHQ-9[1], GAD-7[2], General mood questions	Residents experienced a decrease in mood, increase in anxiety and depression symptoms from the third month of residency (before COVID-19) to the

									sixth month of residency (during COVID-19). These changes were not present in the previous year's cohort.
**Li (2020)(15)**	Cross-sectional survey	Asia (China)	COVID-19	582	Physicians	NR for physicians only	100/0	PHQ-9[1], GAD-7[2], IES-R[6]	Symptoms of stress, depression and anxiety are prevalent in women healthcare workers during the early stage of the COVID-19 pandemic. Women with two or more children or more than 10 years work experience are more likely to experience these symptoms.
**Liu, Q. (2020)(16)**	Qualitative	Asia (China)	COVID-19	4	Frontline	Mean: 11.5<br>Median: 11.5<br>IQR: 7<br>Range: 6-17	25.0/75.0	Categorized interview responses	Working conditions caused increased anxiety. Concerns about ill patients and feel relief when their patients do well and recover.
**Liu, Y. (2020)(17)**	Cross-sectional survey	Asia (China)	COVID-19	858	Pediatric	NR for physicians only	NR for physicians only	DASS-21[5]	Pediatric medical workers in China experience symptoms of depression, anxiety and stress, with men's average stress scores significantly higher than women's. Physicians had a higher average

									perceived stress and depression scores, compared to nurses, as well as a higher prevalence of anxiety and stress.
**Liu, Z. (2020)(18)**	Pre-test post-test (psychological crisis intervention)	Asia (China)	COVID-19	2079	Hospital physicians	NR for physicians only	NR for physicians only	SCL-90[11]	The scores of doctors significantly decreased over the course of the COVID-19 pandemic. Psychological crisis intervention should be carried out early into a pandemic and evaluated for effectiveness.
**Ning (2020)(19)**	Cross-sectional survey	Asia (China)	COVID-19	317	Neurology staff members	Senior: 132 (41.6%) Intermediate: 112 (35.3%) Junior or lower: 73 (23.0%)	49.5/50.5	SDS[12] – Chinese edition, SAS[8]	Symptoms of anxiety and depression are less prevalent in neurology physicians (vs. nurses).
**Que (2020)(20)**	Cross-sectional survey	Asia (China)	COVID-19	860	Various	Education - physicians (years): mean 18.95 (SD 2.17) & residents: 18.37 (SD 1.58)	63.5/36.5	PHQ-9[1], GAD-7[2], ISI[3]	Lowest prevalence of symptoms of anxiety and depression and symptoms of insomnia observed in medical residents
**Shao (2020)(21)**	Cross-sectional survey	Asia (China)	COVID-19	84	Ophthalmologists	Wuhan: Junior College, 2(4.8); Undergraduate, 17 (40.5); ≥Masters, 23 (54.8)	40.5/59.5	HADS[13], VDAS[14]	Ophthalmologists in Wuhan experience more symptoms of anxiety and depression than those in Jiangxi,

						Jiangxi: Junior College, 2(4.8); Undergraduate, 30 (71.4); ≥Masters, 10 (23.8)			but this difference is not significant
**Song (2020)(22)**	Cross-sectional survey	Asia (China)	COVID-19	6,093	Emergency department doctors	NR	NR	PCL-5[15]	Considerable number of emergency department staff experienced symptoms of depression and PTSD
**Wang, H. (2020)(23)**	Cross-sectional survey	Asia (China)	COVID-19	149	NR for physicians only<br><br>Physicians 14.3% of sample. Guangdong province.	NR for physicians only	NR for physicians only	HADS[13] , PSS-14[4], ISI[3]	Less than a quarter of physicians working in Guosong province experienced clinically significant levels of anxiety, depression, stress, and insomnia in the early stages of the COVID-19 pandemic.
**Wang, Y. (2020)(24)**	Cross-sectional survey	Asia (China)	COVID-19	1787	Postgraduate & resident physicians	NR for physicians only	NR for physicians only	K-6[16], IES-R[6], 9-item Chinese version of the Work-Family Conflict and Support Scale[17]	Medical trainees from China who have active clinical duties were at increased risk for psychological distress
**Wu (2020)(25)**	Cross-sectional survey	Asia (China)	COVID-19	14	Anesthesia	NR	50.0/50.0	Multiple choice	Anaesthesiologists with coronavirus have a high prevalence of anxiety that persists through their recovery

**Xu (2020)(26)**	Qualitative semi-structured interviews	Asia (China)	COVID-19	21	Primary care practitioners	3-25 years (mean 12 years)	48/52	Interviews	Providers reported increased fatigue associated with the pandemic, and anxiety related to an increase in tasks and quickly changing protocols.
**Yang (2020)(27)**	Cross-sectional survey	Asia (China)	COVID-19	285	Otolaryngologists	NR for physicians only	NR for physicians only	Survey, SAS[8]	About a quarter of otolaryngologists from this sample in Hubei province, China, self-reported symptoms amounting to anxiety.
**Zhang (2020)(28)**	Cross-sectional survey	Asia (China)	COVID-19	454	Hospital staff including frontline	NR	NR	ISI[3]	Doctors did not report insomnia as much as other groups of health care professionals and medical staff.
**Zhao (2020)(29)**	Prospective cohort	Asia (China)	COVID-19	116	NR	4.36 ± 0.12	59.50/40.5	PSQI[18]	Both physicians and nurses experienced worsening sleep quality over the study period.
**Zhu (2020)(30)**	Cross-sectional survey	Asia (China)	COVID-19	79	Frontline	NR	NR	SAS[8], SDS[12]	Male physicians had a lower chance of showing symptoms of depression compared to females.
**Bajaj (2020)(31)**	Cross-sectional survey	Asia (India)	COVID-19	51	35% Internal medicine, pulmonary medicine, and peadiatric, 25% Para-clinical	NR	36/64	Survey, Likert scale	Doctors in contact with COVID-19 patients are at increased risk of infection and those diagnosed

					specialties, 20% anesthesia, and intensive care				have impact to physical and mental health.
**Banerjee (2020)(32)**	Cross-sectional survey	Asia (India)	COVID-19	133	Otolaryngologists	<5 years to >15 years	NR	Survey	83.5% of otolaryngologists believed COVID19 caused them increased stress which was associated with worry of infecting family and family support if they were to die
**Barik (2020)(33)**	Cross-sectional	Asia (India)	COVID-19	158	Orthopedic residents<br>107 (67.7%) junior residents<br>51 (32.3%) senior residents	NR	NR	Survey	The majority of the residents (51.3%, 53.2%, and 56.3% in Inpatient ward, Operating room, and outpatient department, respectively) were found to be working with anxiety about contracting COVID-19 infection
**Chatterjee (2020)(34)**	Cross-sectional survey	Asia (India)	COVID-19	152	Various	Mean: 17.92<br>SD: 12.1	21.7/78.3	DASS-21[5]	Doctors from India working during the COVID-19 pandemic have a high prevalence of symptoms of depression, anxiety and stress.
**Das (2020)(35)**	Cross-sectional survey	Asia (India)	COVID-19	422	Frontline physicians (188 post-graduate trainees, 126 interns/house staff, 75 senior	NR	44.5/55.5	Survey, PHQ-9[1], PSS[4]	Overall, 63.5% of physicians qualified as having some level of depression and 45% reported

					residents and 33 consultants)				moderate or higher levels of stress. Being female and a post-graduate trainee was significantly associated with increased scores for both depression and stress.
**Gangakhedkar (2020)(36)**	Cross-sectional survey	Asia (India)	COVID-19	707	Anesthesiologist	<5 years to >20 years	52.2/47.8	Survey	Fear of catching the infection and infecting loved ones were important causes of anxiety.
**Gupta (2020)(37)**	Cross-sectional	Asia (India)	COVID-19	749	NR	0 to >20 years (not specific for physicians)	NR for physicians only	Survey, HADS[13]	Approximately one third of the HCWs reported anxiety and depressive symptoms. The risk factors for anxiety were female gender, younger age and job profile and for depressive symptoms were younger age and working at a primary care hospital.<br><br>**aggregate -
**Khanna (2020)(38)**	Cross-sectional survey	Asia (India)	COVID-19	2,355	Trainee ophthalmologists	NR	43.3/56.7	PHQ-9[1]	Ophthalmologists had a high prevalence of symptoms of depression during the COVID-19 pandemic. Symptoms of depression were

									significantly higher at a younger age, with odds of depression decreasing by 3% with each year increase.
**Khasne (2020)(39)**	Cross sectional study	Asia (India)	COVID-19	1667	Frontline	NR for physicians only	NR for physicians only	Self developed survey based on CBI[19]	Frontline physicians working in India during the COVID-19 pandemic experience a high level of pandemic related burnout.
**Mishra (2020)(40)**	Cross-sectional survey	Asia (India)	COVID-19	716	Trainee ophthalmologists	NR	61.6/38.4	Multiple choice	The lockdown resulted in higher stress levels amongst ophthalmology residents, who also felt large changes to their personal routines.
**Pandey (2020)(41)**	Cross-sectional survey	Asia (India)	COVID-19	83	Medical students and Junior doctors	NR	56.6/43.4	Survey, GAD-7[2], PHQ-9[1]	Female students showed higher anxiety and depression than males, but there was no measured deterioration in anxiety and depression for direct patient care and care of patients with COVID-19.
**Podder (2020)(42)**	Cross-sectional survey	Asia (India)	COVID-19	384	Various	Dermatologists: ≤5 yrs: 75.7% 6-9 yrs: 5.6% ≥10 yrs: 18.8% Non-dermatologists:	74.8/25.2	PSS-10[4]	Perceived stress was higher in non-dermatologists (vs. dermatologists). Higher stress was associated with

						≤5 yrs: 88.8%<br>6-9 yrs: 4.2%<br>≥10 yrs: 7.1%			females and unmarried participants.
**Sahu (2020)(43)**	Cross-sectional survey	Asia (India)	COVID-19	611	Orthopaedic surgeons	NR	NR	Multiple choice	22.5% of orthopaedic surgeons were "definitely stressed out" about the COVID-19 pandemic lockdown.
**Sarma (2020)(44)**	Cross-sectional survey	Asia (India)	COVID-19	110	Onco-anesthesia, medical officer, orthodontics, pathology, and physical medicine and rehabilitation	NR	40/60	Survey	Physicians working in non-COVID areas reported stress and challenges related to the effects of the pandemic. Stress related to lack of PPE, education, as well as lack of guidelines and training for patient management. Physicians reported stress, and concern regarding themselves or family contracting the virus. lack of training, concern for lack of PPE, and improper guidelines for patient management were associated with perceived stress.
**Saurabh (2020)(45)**	Cross-Sectional survey	Asia (India)	COVID-19	75	Residents, assistant professors, consultants	NR for physicians only	NR for physicians only	Survey	Physicians were found to report an increase in non-work-related

									stress, as well as stress/concerns regarding preparedness of hospital to deal with COVID-19. Physicians were found to experience worry regarding transmission to family.
**Sil (2020)(46)**	Cross-sectional survey	Asia (India)	COVID-19	41	Frontline dermatologists	Postgraduate: 51.2%<br>Senior residents: 29.3%<br>Consultants: 19.5%	41.5/58.5	PHQ-9[1], PSS-10[4]	Mean perceived stress scores were higher in dermatologists who were female, worked long hours, worked in COVID-positive wards, or stayed away from family.
**Singariya (2020)(47)**	cross-sectional observational study	Asia (India)	COVID-19	512	Anaesthesiologists (Junior, senior residents, junior, senior consultant)<br>Resident n = 350 (68.4%)<br>Consultant n = 162 (31.6%)	NR	44.3/55.7	GAD-7[2], ISI[3]	The majority of anaesthesiologists on COVID19 duty suffer from anxiety and insomnia. Risk factors included being female, being < 35, married, resident, being afraid of infection to self or family, fear of salary deductions, increase in working hours, loneliness due to isolation, food and accommodation issues, and posting in COVID19 duty

**Suryavanshi (2020)(48)**	Cross-sectional survey	Asia (India)	COVID-19	124	66 physicians, 58 residents/ interns	NR for physicians only	NR for physicians only	PHQ-9[1], GAD-7[2]	Physicians and residents/interns reported moderate to severe depression and anxiety, and more so compared to paramedical practitioners, but less compared to nurses.
**Uvais (2020)(49)**	Cross-sectional survey	Asia (India)	COVID-19	58	70.7% working in designated COVID-19 hospitals (Critical care, internal medicine, anesthesia, pediatrics, emergency medicine, gastroenterology & other specialties)	<1 - >10	39.7/58.6	PSS-10[4]	Over 60% of physicians indicated high levels of perceived stigma and stress while working in predominately dedicated COVID-19 hospitals in India.
**Naser Moghadasi (2020)(50)**	Cross-sectional survey	Asia (Iran)	COVID-19	14	Multiple Sclerosis Fellowships	NR	50/50	BAI[20]	Only 2 participants had a mild level of anxiety.
**Vafaei (2020)(51)**	Cross-sectional survey	Asia (Iran)	COVID-19	194	Obstetrics/gynecology specialist (residents are not separated from med students)	NR	NR	PHQ-9[1], MSPSS[7], SF-36[21]	Obstetrics and gynecology specialists practicing in Iran during the COVID-19 pandemic experienced symptoms of anxiety, but had significantly higher social functioning and general health scores compared to residents/students or nurses/midwives

**Abdulah & M. A. (2020)(52)**	Cross-sectional survey	Asia (Iraqi Kurdistan)	COVID-19	209	Pediatrics, emergency, specialized Coronavirus, maternity and gynecology hospitals	0-42 years	25.4/74.6	PSS-10[4]	Medical doctors at a variety of hospitals in Iraqi Kurdistan experienced a moderate level of perceived stress during the COVID-19 outbreak in the region. Perceive stress during infection outbreaks can lead to mental issues like stress, anxiety, fear and depression which can have devastating impacts on healthcare workers. These issues can also impact decision=making, attention and understanding for healthcare workers which is critical for the wellbeing of both patients and practitioners.
**Abdulah & M. D. (2020)(53)**	Cross-sectional study	Asia (Iraqi Kurdistan)	COVID-19	268	Anesthesia and intensive care, lab specialties, internal medicine specialties, community and family medicine, surgery, general physicians (GP), obstetrics and gynecology,	mean: 10.13 years	29.9/70.1	AIS[22]	The study found that assisting COVID-19 patients negatively affected the sleep of physicians. 68.3% of physicians were sleepless and 93.7% had stress. There was no significant

					pediatrics, psychiatry, and radiology				difference between the sleep scores of physicians in different specialties. The physicians who handled suspected or confirmed COVID cases had escalated sleep similar to those experiencing stress. This was notably different compared to physicians who did not deal with COVID patients or experience stress. As the number of days physicians handled COVID patients increased, their sleep also escalated.
**Milgrom & R. (2020)(54)**	Cross-sectional survey	Asia (Israel)	COVID-19	45	Internal medicine residents	NR	33.6/66.7	STAI-S[23]	Internal medicine residents at a COVID-19 treatment center were more concerned about the possibility of infecting their families (vs COVID-19 free hospital)
**Milgrom & T. (2020)(55)**	Cross-sectional survey	Asia (Israel)	COVID-19	337	Hospital physicians	NR	NR for physicians only	STAI-S[23]	Highest anxiety scores were among resident doctors and the lowest among senior doctors. Resident doctors are at increased

									risk (OR 2.1, 95%CI: 1.4-3.2) of clinical anxiety compared to other hospital staff
**Mosheva (2020)(56)**	Cross-sectional survey	Asia (Israel)	COVID-19	1106	Physicians	NR	49/51	PROMIS[24], CD-RISC[25], PRSF inventory[26]	There is an associated between high anxiety scores and mental exhaustion, anxiety about being infected, anxiety infecting family members, and sleep difficulties. Conversely, resilience is associated with lower anxiety scores.
**Awano (2020)(57)**	Cross-sectional Survey	Asia (Japan)	COVID-19	104	Frontline	NR	76.0/24.0	GAD-7[2], CES-D[27] and 10-item CD-RISC[25], and original questionnaire	The median anxiety score for all participants was 4. Proportionally, 90.0% of participants had no or mild anxiety and 10.0% had moderate-to-severe anxiety. For depression, the median CES-D score was 12 and 27.9% of healthcare workers developed symptoms of depression. The median resilience score was 22 (IQR: 18-27). For the original questionnaires,

									doctors had lower scores for regarding anxiety and fear of infection and death compared to other healthcare workers. Younger workers had higher scores on this questionnaire than their older colleagues. After dividing the participants into a healthy group and a group with depression, researchers found that those in the depression group were more likely to experience moderate-to-severe anxiety. The resiliency score for that group was also a lot lower than it was for the healthy group.
**Naser (2020)(58)**	Cross-sectional survey	Asia (Jordan)	COVID-19	560	Various	NR	NR	PHQ-9[1], GAD-7[2]	Frontline physicians experienced more symptoms of depression and anxiety.
**Saadeh (2020)(59)**	Cross-sectional survey	Asia (Jordan)	COVID-19	135	Frontline, general physicians providing direct care to COVID-19 patients	NR	34.1/65.9	Survey, Likert scale	Fear was reported by more than half of physicians in the study. The majority of physicians expressed concern

									of contracting COVID-19 themselves and transmitting to families. Physicians expressed concerns regarding long-term impact on health care services of COVID-19.
**Mohd Fauzi (2020)(60)**	Cross-sectional survey	Asia (Malaysia)	COVID-19	1050	Medical doctors working at all government health facilities	NR	71.5/28.5	survey, NASA-TLX[28], OFER-15[29], DASS-21[5], modified REQ[30]	Majority of Malaysian doctors working in government health facilities has no symptoms of depression, anxiety or stress. Adverse mental health symptoms were associated with higher work demands and lower recovery.
**Amin (2020)(61)**	Cross-sectional survey	Asia (Pakistan)	COVID-19	389	Frontline	NR	48.3/51.7	SRQ-20[31]	43% of participants were flagged for depression/anxiety. Physicians who were assessing more than 5 COVID suspects/patients per day were more at risk along with participants who were working 20 hours/week or less, those who had children living with them, and providers who had

									moderate to low knowledge of the infection.
**Arshad (2020)(62)**	Cross-sectional Survey	Asia (Pakistan)	COVID-19	431	Frontline	NR	44.78/55.2	GAD-7[2]	Mild, moderate and severe anxiety was seen in 27.8%, 23.9% and 9.7% of doctors, respectively. The median anxiety score Median score was 6.
**Ayub (2020)(63)**	Cross-sectional survey	Asia (Pakistan)	COVID-19	208	Frontline	NR	66.3/33.7	Online questionnaire	83.7% of respondents were reluctant to treat patients with COVID-19. The most common concerns related to this were: a lack of PPE (92% feared this), fears of getting sick themselves, excessive workload, and fear they would spread the virus to their loved ones (74% stated this fear). 67.3% were afraid that their own anxiety was impacting their ability to do their job. In terms of possible solutions, 93.3% of participants stated that psychological counselling should be provided and 96.2% felt that

									hospitals should provide staff with places to rest and isolate in order to avoid spreading the virus to their families.
**Hasan (2020)(64)**	Cross-sectional survey	Asia (Pakistan)	COVID-19	151	NR	NR	56.3/43.7	GAD-7[2]	Doctors dealing with COVID-19 patients showed significantly higher levels of anxiety compared to doctors who were not caring for COVID-19 patients.
**Imran (2020)(65)**	Cross-sectional survey	Asia (Pakistan)	COVID-19	10178	Postgraduate residents (medicine, surgery and basic science)	1-4 years	56.7/ 43.3 (Gender)	PHQ-9[1], GAD-7[2], SASRQ[32]	The prevalence of depressive symptoms, generalized anxiety disorders and acute stress disorder were 26.4%, 22.6%, 4.4%. Female, postgraduate trainees, senior trainees and front-line workers reported experiencing significantly more anxiety, depression and acute stress symptoms ($p<0.001$). Being front line staff and female was correlated with higher risk of experiencing symptoms of depression,

									anxiety and acute stress.
**Osama (2020)(66)**	Cross-sectional survey	Asia (Pakistan)	COVID-19	112	Surgical residents	1st year: (15.2%)<br>2nd year: 21 (18.8%)<br>3rd year: 25 (22.3%)<br>4th year: 25 (22.3%)<br>5th year: 24 (21.4%)	40.2/59.8	Modified MBI[33], survey	Surgical residents from Pakistan experienced the fear of being infected by COVID-19
**Rashid (2020)(67)**	Qualitative (IPA)	Asia (Pakistan)	COVID-19	13	Resident physicians	NR	31/69	Interviews	Young doctors experienced psychological distress, and emotional vulnerability due to the challenges and concerns faced by them in the wake of COVID-19. Shortage and excessive reuse of personal protective equipment, direct exposure to the disease, concern for personal safety, fear and apprehension of being a probable disease carrier for families, physical distancing from loved ones, long working hours, and increased organizational responsibility altered the quality of life for doctors

									working frontline toward pandemic in COVID wards. Doctors who received appreciation and support by families reported improved filial bonds.
**Urooj (2020)(68)**	Cross-sectional survey	Asia (Pakistan)	COVID-19	222	Various	NR	67.5/32.5	Multiple choice	Physicians experience fears related to the wellbeing of their families and not being provided appropriate PPE during the COVID-19 pandemic
**Sorokin (2020)(69)**	Cross-sectional survey	Asia (Russia)	COVID-19	1512 *(84% of 1800)	Frontline - Anesthesiologist/ ICU physicians; Epidemiology & infectious diseases; Internal medicine, pulmonary, and GP; Other medical specialties & psychiatrist	NR for physicians only	NR for physicians only	PSM-25[34]	Physicians were more likely to indicate higher levels of stress compared to other healthcare workers, with anesthesiologists and ICU physicians indicating higher levels than other physicians and HCWs.
**Al Sulais (2020)(70)**	Cross-sectional survey	Asia (Saudi Arabia)	COVID-19	529	Various	NR	59.2/40.8	Likert scale questions	Physicians commonly reported during the COVID-19 pandemic feelings of worry, isolation and fear. Older physicians (>60 years) were less likely to feel isolated and female physicians were more likely

									to experience fear or worry. Physicians with past pandemic experience were less likely to experience fear.
**Alahmadi (2020)(71)**	Cross-sectional	Asia (Saudi Arabia)	COVID-19	142	Ophthalmology residents	Level of training Ophthalmology residents R1, n = 51, 27.9% R2, n = 46, 25.1% R3, n = 43, 23.5% R4, n= 43, 23.5% Survey participants R1, n = 45, 31.7% R2, n = 27, 19.0 % R3, n = 38, 26.8% R4, n= 32, 22.5%	Ophthalmology residents 42.1/57.9 Survey participants 39.4/60.6	PHQ-9[1]	COVID-19 had a negative impact on trainees' mental health.
**AlAteeq (2020)(72)**	Cross-sectional survey	Asia (Saudi Arabia)	COVID-19	111	NR	NR	31.9/68.1	PHQ-9[1], GAD-7[2]	This study showed that mental health issues like depression and generalized anxiety are affecting many healthcare workers. Additional attention and support needs to be provided, particularly for female providers, those between the

									ages of 30-39, and nurses who were shown to struggle most with these conditions.
**Almater (2020)(73)**	Cross-sectional survey	Asia (Saudi Arabia)	COVID-19	107	Residents, fellows, registrars, consultants	NR	43.9/56.1	PHQ-9[1], GAD-7[2], ISI[3], PSS-10[4]	Symptoms of depression were exhibited by 50.5% of participants. Anxiety was exhibited by 46.7%. Insomnia was exhibited by 44.9%. Low stress symptoms were shown by 28% of the population, 68.2% experienced moderate symptoms and 3.7% experienced symptoms of high stress. In severe cases 29% of participants were identified as having depression, 38.3% had anxiety, and 15% had insomnia. Female ophthalmologists were more likely to experience depression, as well as those living with someone elderly. Fellows were also more likely to experience depression. Female ophthalmologists were also more likely to struggle

									with anxiety and to experience more moderate-to-high symptoms of stress.
**Alnofaiey (2020)(74)**	Cross-sectional survey	Asia (Saudi Arabia)	COVID-19	369	Residents, fellows, registrars, consultants	NR	50.9/49.1	PSQI[18]	43.9% of participants had a sleep disorder. Researchers found that COVID-19 has had a negative impact on the quality of sleep obtained by HCWs
**Chew (2020)(75)**	Cross-sectional	Asia (Singapore)	COVID-19	274	Residents medical (medical specialties, family medicine, radiology) surgical disciplines (surgery, anaesthesia, emergency medicine), and psychiatry<br><br>Junior (61.7%) & senior (38.3%)	Cohort was split into residents in junior (1st 3 years) and senior years (4th year onwards) of training	Not reported separately from the analyses	PSS[4], IES-R[6], HWSS[35] Brief COPE[36]	Residents form a sizable proportion of frontline healthcare workers in the pandemic, initiatives to raise awareness of psychological and coping responses, emphasize self-care, address issues of stigma, and provide access to resources for help can enhance psychological support for our residents in training. Those deployed to high risk areas had lower PSS scores than those who were not deployed which authors conclude relates to the level of psychological

									preparedness in those deployed
**Lee (2020)(76)**	Cross-sectional survey	Asia (Singapore)	COVID-19	270	Anaesthesiologists	NR for physicians only	NR for physicians only	GHQ-12[37], HADS[13]	Anaesthesiologists working in ICUs experience significant psychological distress.
**Tan (2020)(77)**	Cross-sectional survey	Asia (Singapore)	COVID-19	NR	Surgeons	NR	NR	ProQOL-5[38]	Physicians were less likely to fear contracting COVID-19 and spreading COVID-19 than other healthcare provider, although the prevalence of these fears was still high. Physicians were less likely to report burnout and secondary traumatic stress compared to non-physicians.
**Chang (2020)(78)**	Cross-sectional	Asia (South Korea)	COVID-19	229	Orthopedic residents	gr 1 - 54, 23.6%<br>gr 2 - 60, 26.2%<br>gr 3 - 48, 21.0%<br>gr 4 - 59, 25.8%<br>No answer - 8, 3.4%	3.1/95.6/1.3 (No answer)	Survey	The average score for quality of life, which was 68.9 out of 100 scores before the pandemic, decreased to 61.7 during the pandemic ($p < 0.001$). The most stressful factor for orthopaedic residents during the pandemic was family/relative health, followed by their own health and

									residency program.
**Jo (2020)(79)**	Cross sectional survey	Asia (South Korea)	COVID-19	27	Frontline	Not reported	41/59	Korean version of IES-R[6], MINI[39], CGI-S[40]	Nurses and auxiliary staff had significantly higher IES-R scores than the physicians. Both the IES-R and CGI-S scores of high-risk participants had decreased significantly upon second evaluation after two weeks.
**Al Mahyijari (2020)(80)**	Cross-sectional survey	Asia (Sultanate of Oman)	COVID-19	59 Physicians	Frontline	Mean: 13.78 years	50.1/49.9	PSS[4], GAD-7[2], WHO-5[41]	The study found that a significant number of healthcare professionals experienced high levels of stress and anxiety. They also found that workers were experiencing suboptimal levels of wellbeing which was connected to perceived stress and anxiety. This study supports other literature that has highlighted the negative impact of COVID-19 on the wellbeing of frontline healthcare professionals
**Kuo (2020)(81)**	Cross sectional study	Asia (Taiwan)	COVID-19	54	NR	9.9 years on average (+-7.9	31.5/68.5	Healthcare Workers' Stress	Hospital staff reported a moderate level of

						years) range 1-41 years		Related to Caring for Patients with a Highly Infectious Disease Scale[42]	stress. Participants who experience severe stress, work stress was higher among those with minor children compared to those without.
**Caliskan (2020)(82)**	Cross-sectional survey	Asia/Europe (Turkey)	COVID-19	290	Emergency physicians	1 to >15	38.3/61.7	HADS[13]	62% and 35.5% of emergency physicians experienced depression and anxiety, respectively, during the COVID-19 pandemic, and those who were well informed, received COVID-19 training and had COVID-19 protocols were less likely to experience these symptoms
**Elbay (2020)(83)**	Cross-sectional survey	Asia/Europe (Turkey)	COVID-19	442	Various	NR	56.8/43.2	DASS-21[5]	Physicians in Turkey had high prevalence of symptoms of depression, anxiety and stress during the COVID-19 pandemic. Being female, early career, young and single, or working on the frontline were associated with more symptoms of depression, anxiety and stress.

**Hacimusalar (2020)(84)**	Cross-sectional survey	Asia/Europe (Turkey)	COVID-19	471	NR	172.8 +/- 109.9 months	NR for physicians only	STAI[23], BHS[43]	Healthcare workers were more affected psychologically in the COVID-19 pandemic compared to society.
**Korkmaz (2020)(85)**	Cross sectional study	Asia/Europe (Turkey)	COVID-19	30	Frontline	<1 year: 3(10%)<br>1 to 5 years: 4 (13%)<br>>5 years: 23 (77%)	33/67	BAI[20], WHOQOL-BREF[44], PSI[45], PSQI[18]	Clinically significant anxiety was found in 33% of study participants. The number of participants without anxiety was 41 (295), with mild anxiety 53 (38%). PSQI and PSI scores of nurses were significantly higher compared to physicians and other staff whereas WHOQOL-BREF scores were lower compared to physicians and other staff.
**Kurt (2020)(86)**	Cross-sectional survey	Asia/Europe (Turkey)	COVID-19	264	Internal & surgical trainees	Median 2.6 (SD 1.2) years (assistantship duration)	51.9/48.1	STAI[23], BDI[46]	Prevalence of symptoms of anxiety and depression higher in female physicians (vs. male physicians). Fear of being infected by COVID-19 (or infecting others) is associated with anxiety.

**Sahin (2020)(87)**	Cross-sectional survey	Asia/Europe (Turkey)	COVID-19	580	frontline	NR for physicians only	NR for physicians only	PHQ-9[1], GAD-7[2], ISI[3], IES-R[6]	Physicians found to be experiencing greater depression, anxiety, insomnia and distress through the COVID-19 pandemic. Higher levels of these psychological symptoms reported among those who had to be tested for COVID-19, females, those working frontline, and those with psychiatric illness in their health history.
**Saracoglu (2020)(88)**	Cross-sectional survey	Asia/Europe (Turkey)	COVID-19	34	Anesthesiologists	NR for physicians only	NR for physicians only	PHQ-9[1], PSQI[18], FCV-19S[47]	Physicians were found to be experiencing depression and fear, particularly those working in intensive care units. Younger age associated with more COVID-19 reported fear, while advanced age more fear related to loss of life.
**TaŞ (2020)(89)**	Cross-sectional survey	Asia/Europe (Turkey)-	COVID-19	448	Family physicians	0-3 years: 76/448 (17%)<br>3-10 years: 124/448 (27.7%)	43.3/56.7	Job Strain Scale Short Form[48], questionnaire	Family Physicians practicing in Istanbul during the COVID-19 pandemic experienced a decrease in sleep

						10-30 years: 216/448 (48.2%)<br>30 years and over: 32/448 (7.1%)			quality and increase in symptoms of anxiety
**Uyaroglu (2020)(90)**	Cross-sectional survey	Asia/Europe (Turkey)	COVID-19	113	77 Residents, 36 academic staff. 63.1% of total working frontline	2.3 years (SD 2.1) & academic staff 7(SD18)	46.9/53.1	GAD-7[2], BAI[20]	Academic staff and residents did not have significant differences in anxiety scores. Being female, having family members over 65 years old, having a chronic disease were significantly associated with higher anxiety scores.
**Yalçın Bahat (2020)(91)**	Cross-sectional survey	Asia/Europe (Turkey)	COVID-19	253	Obstetricians & gynecologists (frontline)	Consultants: 12.3%<br>Residents: 87.7%	54.4/45.6	Multiple choice	Most OB/GYNs surveyed indicated levels of stress related to different factors of the pandemic such as concern for their family and the possibility of infecting them.
**O'Kelly (2020)(92)**	Cross-sectional survey	Asia/Europe /North America	COVID-19	58	Pediatric urologists	<5 years to >15 years	39.7/61.3	Survey, PSS[4], Discussions	COVID-19 has impacted paediatric urologists in the following ways: decreased number of patients seen and operated on, decreased salary, increased personal stress levels, substantially increased telemedicine,

									increased free time for various activities and good compliance with guidelines and hospital management decisions.
**Degraeve (2020)(93)**	Cross-sectional survey	Europe (Belgium)	COVID-19	62	Urology residents	Year of training of training<br>2 (24.2%)<br>3 (24.2%)<br>4 (25.8%)<br>5 (8.1%)<br>6 (17.7%)	NR	Survey, CBI[19]	The COVID-crisis had a positive impact on both personal life and theoretical training for urology residents. Burnout risk scores were decreased due to the COVID-19 crisis.
**Salopek-Ziha (2020)(94)**	Cross-sectional survey	Europe (Croatia)	COVID-19	27	Frontline and general physicians (15% off all participants worked directly with COVID-19 patients)	NR	NR	SF-36[21], DASS-21[5], WOC[49]	Physicians had lower mean depression and anxiety scores than nurses. Doctors were found to use less avoidant coping styles and positive reappraisals than nurses.
**Abdessater (2020)(95)**	Cross-sectional survey	Europe (France)	COVID-19	275	Junior & senior residents	Urology residents (%):<br>1st year (8)<br>2nd year (12)<br>3rd year (11)<br>4th year (11)<br>5th year (15)	33/67	Likert scale questions	The COVID-19 pandemic impacts urology residents, with 90% of responders feeling more stressed by the pandemic and more than half reporting medium to high level of stress. Risk factors for stress included COVI-19 patients in their

									department, medical history of respiratory disease or caring for patients with COVID-19.
**Florin (2020)(96)**	cross-sectional	Europe (France)	COVID-19	1515	Radiologists Junior (resident or fellow) (n:397, 26.2%) Academic radiologist (n:85, 5.6%) Non-academic hospital radiologist (n: 224, 14.8%) Private practice radiologist (n: 809, 53.4%)	NR	44.3/55.7	ISI[3], HADS[13]	Among responders, 186 (12.3 %) expressed insomnia, 222 (14.6 %) anxiety, and 189 (12.5 %) depression symptoms. Lack of protective equipment, increased teleradiology activity and negative impact on education were risk factors for insomnia. Female gender, respiratory history, working in COVID-19 high density areas, increased COVID-19 related activity and impacted education were risk factors for anxiety. Working in a public hospital was a protective factor against insomnia, anxiety, depression
**Hilmi (2020)(97)**	Cross sectional survey	Europe (France)	COVID-19	222	Oncology & radiation therapy residents	NR	56/44	Survey, HADS[13]	French oncology residents were highly affected by the first peak of COID-19 pandemic

									in terms of professional activity and psychological impact.
**Vallee (2020)(98)**	Cross-sectional survey	Europe (France)	COVID-19	1001	Residents and fellows	NR	51.6/48.4	GAD-7[2], PHQ-9[1], ISI[3]	Residents and fellows reported anxiety, depression, and insomnia, with levels higher in women and those who use alcohol or tobacco. Sufficient PPE and training were protective factors.
**Bohlken (2020)(99)**	Cross-sectional survey	Europe (Germany)	COVID-19	396	Psychiatrists & neurologists	Mean: 56.9 SD: 7.589	41.7/58.3	Likert scale questions	Even though 91% of psychiatrists and neurologists did not have contact with patients, 18% reported substantial anxiety and 9% reported sleep problems.
**Büntzel (2020)(100)**	Cross-sectional survey	Europe (Germany)	COVID-19	47	Oncologists	NR	NR	Likert scale questions	Physicians have some concern for themselves and their health, as well as fears about the health of their patients.
**Kramer (2020)(101)**	Cross sectional study	Europe (Germany)	COVID-19	1618	Emergency, ward, COVID-19-unit, ambulatory care, ICU	NR	42.9/57.1/0.1 (Third)	Likert scale questions	Nurses had significantly higher subjective burden and subjective stress than MDs and other staff. MDs reported lower values on stress and

									subjective burden than nurses and other staff.
**Skoda (2020)(102)**	Cross-sectional survey	Europe (Germany)	COVID-19	492	Physicians	NR	65.7/34.2/0.2 (Divers)	GAD-7[2], PHQ-2[1], EuroQol EQ-5D-3L[50], survey	German physicians show less generalized anxiety and depression scores than nursing staff. High subjective level of information regarding COVID-19 is associated with less psychological burden.
**Zerbini (2020)(103)**	Cross-sectional survey	Europe (Germany)	COVID-19	35	University hospital	NR	NR	Multiple choice	Physicians in COVID-19 wards experiences similar symptoms of stress, exhaustion, and depression compared to their colleagues in regular wards
**Diomidous (2020)(104)**	Cross-sectional	Europe (Greece)	COVID-19	102	NR	NR	71.3/28.7	AIS[22], Jenkins Sleep Scale[51], Sleep_50 Questionnaire[52]	There were positive correlations between physical activity during daily work and free time with parameters related to sleep disorders during the COVID-19 pandemic
**Farooq (2020)(105)**	Cross-sectional	Europe (Ireland)	COVID-19	285	Doctors in training	NR	NR	PSS[4]	Majority of junior doctors had moderate stress at baseline. They reported concerns

									regarding their personal and family health as well as impact on social life
**Amerio (2020)(106)**	Cross-sectional survey	Europe (Italy)	COVID-19	131	General practitioners	NR	48.1/51.9	PHQ-9[1], GAD-7[2], ISI[3], SF-12[21]	General practitioners reporting moderate to severe depressive symptoms reported more helplessness and visited more COVID-19 infected patients. GPs with at least moderate symptoms of depression had higher severity of anxiety and insomnia and worse mental quality of life.
**Bitonti (2020)(107)**	Cross-sectional	Europe (Italy)	COVID-19	933	Residents in obstetrics and gynecology	Collected year of residency but not reported	81.3/18.7	Survey	More than half of the Residents reported anxiety related to fear of contagion, during invasive and non-invasive procedures. Almost all the residents (457/476, 96 %), reported that COVID-19 had negative psychological impact in terms of changes in mood, of which 10 % had their mood totally impacted and 58 %

									considerably impacted by the pandemic.
**Buselli (2020)(108)**	Cross-sectional survey	Europe (Italy)	COVID-19	85	NR	NR for physicians only	NR for physicians only	ProQOL-5[38], PHQ-9[1], GAD-7[2]	Symptoms of burnout, depression and anxiety were reported by physicians during the COVID-19 outbreak
**De Sio (2020)(109)**	Cross-sectional survey	Europe (Italy)	COVID-19	695	Primary care, hospital, freelance	NR	45.5/54.5	Survey, GHQ-12[37], WHO-5[41]	Italian doctors working in the most affected regions of the country experience psychological distress and poor well-being.
**Di Monte (2020)(110)**	Cross-sectional survey	Europe (Italy)	COVID-19	102	General practitioners	< 3: 7.8%<br>3-5 years: 2%<br>5-10 years: 3.9%<br>>10 years: 86.3%	62.7/36.3/1 (other)	MBI[33], RS-14[53], IU-S[54], CISS[55]	Italian general practitioners experienced burnout during the COVID-19 pandemic. Implementing task-oriented coping strategies (as opposed to emotional strategies) appears to protect against burnout.
**Vanni (2020)(111)**	Cross-sectional survey	Europe (Italy)	COVID-19	35	Physicians, surgeons	NR	NR for physicians only	DASS-21[5], PSS[4]	There were no significant differences in the self-reported levels of depression, anxiety, or stress.

**Gallopeni (2020)(112)**	Cross-sectional survey	Europe (Kosovo)	COVID-19	253	NR	NR	NR for physicians only	HADS[13]	Females were more likely to experience depressive symptoms and anxiety symptoms. Physicians had lower odds for depressive and anxiety symptoms compared to other professionals.
**Johnson, S. (2020)(113)**	Cross-sectional survey	Europe (Norway)	COVID-19	178	Physicians	NR for physicians only	NR for physicians only	PCL-5[15], GAD-7[2], PHQ-9[1], items from the following scales: CAS-1[56], IIP[57]	Health works who work directly with COVID-19 patients have significantly higher levels of PTSD symptoms and depression
**Rajwa (2020)(114)**	Cross-sectional survey	Europe (Poland)	COVID-19	229	Urologist (residents)	NR	13.5/84.5	Survey	Most urologists practicing in Poland during the COVID-19 pandemic reported that COVID-19 impacted their clinical practice and harmed their training. Moreover, they experienced symptoms of anxiety, sadness and stress.
**Rymarowicz (2020)(115)**	Cross-sectional survey	Europe (Poland)	COVID-19	304	General surgeons	NR	30.6/69.4	Survey, Numeric scales	A high number of surgeons disclosed fear of working through the COVID-19 outbreak. Reporting demographics/ factors of younger

									age, female sex, having children, being a current trainee, working in a district hospital, and lower self-assessed knowledge about COVID-19 pandemic impacted psychological symptoms.
**Dimitriu (2020)(116)**	Cross-sectional survey	Europe (Romania)	COVID-19	100	Residents (various)	NR	58.0/42.0	MBI[33]	Symptoms of burnout was more prevalent in medical residents working in normal wards (86%) versus medical residents working in frontline departments (66%)
**Guillen-Astete (2020)(117)**	Cross-sectional survey	Europe (Spain)	COVID-19	328 (195 family doctors + 69 internists = numbers do not add up).	Emergency physician (translation maybe skewed - possibly included family & internists as well)	NR	75.3/24.7	BAI[20], HAM-D[58] (translated)	Younger physicians show higher levels of anxiety.<br><br>**MA - potentially an additional finding - An increase in demands on emergency physicians during COVID-19 associated with significant reports of anxiety and depression.
**Martinez-Lopez (2020)(118)**	Cross-sectional survey	Europe (Spain)	COVID-19	22	Physicians	NR for physicians only	NR for physicians only	MBI[33]	Spanish physicians have a higher risk of suffering from emotional

									exhaustion than other professions (OR: 4.3, 95%CI: 1.2-14.5)
**Ruiz-Fernandez (2020)(119)**	Cross-sectional survey	Europe (Spain)	COVID-19	108	NR	NR	NR for physicians only	ProQoL[38], PSS-14[4]	Compassion fatigue and burnout was higher in physicians than nurses. Both professions displayed similar perceived stress scores. Working in a COVID-19 unit or emergency department led to higher compassion fatigue and burnout in both professions.
**Coyle (2020)(120)**	Cross-sectional survey	Europe (United Kingdom)	COVID-19	2075	92% Medical students and 8% newly qualified doctors	NR	NR	Survey	The mean mood score of participants on a scale of 0 to 100 was 51.8. Exercise was a factor in improved mental well-being.
**Faderani, (2020)(121)**	Cross-sectional survey	Europe (United Kingdom)	COVID-19	172 redeployed doctors	72 Foundation trainees (42%), 45 core/ specialty trainees 1–3 (24%), 39 higher specialty trainees 4–7 (23%) and 19 consultants (11%)<br><br>59% Dedicated COVID19 ward, 24% Non-clinical ward, 3.9% ITU, 10.7% General	NR	NA	Questionnaire & free text comments	54.7% did not feel safe while wearing PPE. The three most common concerns were training opportunities, PPE and family health

					non-COVID19, 5.9% Emergency medicine, 18.0% Acute internal medicine				
**Norton (2020)(122)**	Cross-sectional survey	Europe (United Kingdom)	COVID-19	2075	Physicians (interim Foundation year 1 (FiY1) and medical students) (Note: It looks like FiY 1 doctors are residents)	Up to 1 year of post-graduate work	74.5/25.1/0.4 (Non-binary)	Survey, Likert scale	COVID-19-related anxiety was significantly higher in those without sufficient reported PPE or infection prevention and control (IPC) training, in women compared with men, and in FiY1 doctors compared to medical students.
**Payne (2020)(123)**	Cross-sectional survey	Europe (United Kingdom)	COVID-19	32	Surgical trainees, foundation year 2 doctors (FY2) and junior clinical fellows	Post-graduate years 2 to 4	41/59	Survey, Likert scale	Redeployment of surgical trainees to ICU led to increased confidence in technical and non-technical skills. However, mental health deteriorated in this population.
**Rimmer (2020)(124)**	Unclear	Europe (United Kingdom)	COVID-19	6610	NR	NR	NR	Survey	More than two fifths of doctors in the UK say that their mental health is now worse than before the pandemic
**Shah (2020)(125)**	Cross-sectional survey	Europe (United Kingdom)	COVID-19	207	OBGYN	Consultants: 58 (28%) Specialty registrars: 99 (47.8%)	81.1/18.9	Survey, GAD-2[2], PHQ-2[1]	OBGYNs reported higher rates of MDD and GAD compared to the UK estimates. Female doctors

						Senior House Officers: 50 (24.2%)			were more anxious than male doctors.
**De Wit (2020)(126)**	Prospective cohort - surveys	North America (Canada)	COVID-19	468	Emergency department physicians (91%) and trainees (9%)	NR	49/51/1 (Non-binary)	Survey, MBI[33]	Burnout levels across the study period did not significantly vary.
**Gill (2020)(127)**	Cross-sectional survey	North America (Canada)	COVID-19	159	Oncologists	< 5 yrs: 25%<br>5-10 yrs: 19%<br>10-15 yrs: 15%<br>>15yrs: 41%	NR	Multiple choice	Most (70%) oncologists report concerns about contracting COVID-19, and concerns about their family or patients contracting COVID-19 from them
**Odedra (2020)(128)**	Cross-sectional survey	North America (Canada)	COVID-19	96	Radiology residents	PGY1: 15.6%<br>PGY2: 16%<br>PGY3: 25%<br>PGY4: 17.5%<br>PGY5: 25%	NR	Multiple choice	Canadian residents reported an overall stress of exposure to disease was moderate to low, with 13% reporting high or extreme overall stress.
**Pilar (2020)(129)**	Cross-sectional survey	North America (Canada)	COVID-19	15	Radiology oncology fellows	NR	NR	Survey (5-point Likert scale), modified OBI[59]	Radiology fellows in Canada experienced anxiety, burnout, and had difficult sleeping at night during the COVID-19 pandemic in addition to concerns about their learning. Good leadership and their focusing on learning, social interactions and patient care gave them the ability to cope

**Civantos (2020)(130)**	Cross-sectional survey	North America (United States)	COVID-19	349	Otolaryngology residents	NR	39.3/60.7	PHQ-2[1], GAD-7[2], IES[6], Mini-Z[60]	During the COVID-19 pandemic, otolaryngologists physicians had a high prevalence of burnout, anxiety, distress and depression. Attendings had less burnout than residents. Females had increased burnout, anxiety and distress.
**Collins (2020)(131)**	Cross-sectional survey	North America (United States)	COVID-19	73	Surgical residents	Intern: 32 (43.8) Junior resident: 21 (28.8) Senior resident: 20 (27.4)	56.2/42.5/1.4 (Prefer not to say)	GAD-7[2], Survey	Surgical residents experience anxiety about contracting COVID-19 but are more concerned about their loved ones contracting COVID-19. Surgical residents were more likely to have higher GAD-7 scores compared to interns. Senior residents had disproportionately higher GAD-7 scores (suggested to be related to impending professional development) compared to other residents/interns. Senior residents were 10.11 times more likely to have a high GAD-7 score than the interns

**Demirjian (2020)(132)**	Cross-sectional survey	North America (United States)	COVID-19	689	Radiologists	NR	47/53	Survey, Likert scale	Over half of radiologists had increased anxiety as a result of the COVID-19 pandemic. Family health, personal health and financial concerns were leading cause for anxiety. Radiologists that did not need coping strategies reported lower anxiety.
**Fargen (2020)(133)**	Cross-sectional	North America (United States)	COVID-19	151	Radiology (n=56, 37.1%) Neurology (n=41, 27.2%) Neurosurgery (n=54, 35.8%)	less than 5yrs (n = 33, 22.2%) 5-9yrs (n = 36, 24.2%) 10-19yrs (n = 53, 35.6%) More than 20yrs (n = 27, 18.1%)	13.9/86.1	Survey	Sixty-six percent of respondents reported increased career stress, 56% increased personal life/family stress, and 35% increased career burnout. Stress was significantly increased in physicians with COVID-positive family members ($P<0.05$).
**Fitzpatrick (2020)(134)**	Prospective survey study	North America (United States)	COVID-19	55	Emergency Physicians Male: Resident Physicians (n=17) & Attending physicians (n=22) Female: Resident Physicians (n=6) & Attending Physicians (n=10)	NR	29.0/70.9	Survey	EPs felt less in control (p-value = 0.001); felt decreased happiness while at work (p-value 0.001); had more trouble falling asleep (p-value = 0.001); had an increased sense of dread when thinking of work

									needing to be done (p-value = 0.04); felt more stress on days not at work (p-value <0.0001); and were more concerned about their own health (p-value <0.0001) and the health of their families and loved ones (p-value <0.0001).
**Huffman (2020)(135)**	Cross-sectional survey	North America (United States)	COVID-19	Prose says 720 but table 1 says 662	Most are internal medicine (n=187), pediatrics (n=157) and anesthesia (n=102). Most (n=403) are attendings or residents (n=154)	0 to >20 years	NR	Survey, Grit-S[61], CD-RISC[25]	Respondents rated their stress to be significantly increased during the pandemic, with fear of transmitting the virus to their family members being reported as a significant stressor. Healthcare workers have increased resilience in the face of heightened stress during a pandemic. Higher resilience and grit were protective factors in managing personal and system level stressor at the peak of the COVID-19 pandemic.
**Jha (2020)(136)**	Cross sectional survey	North America	COVID-19	100	Pain physicians	< 5 years: 8 (8%)	19/81	Survey	98% of respondents said that their

		(United States)				5-10 years: 13 (13%)<br>10-15 years: 18 (18%)<br>15-20 years: 16 (16%)<br>20-30 years: 36 (36%)<br>>=31 years: 9 (9%)			operations have been impacted by COVID-19. 67% of physicians responded that in house billing was responsible for increased level of burnout, 73% responded that responded that electronic medical records were one of the causes.
**Johnson, A. (2020)(137)**	Cross-sectional survey	North America (United States)	COVID-19	145	Vascular surgeon trainees	PGY 1-2: 23 (15.9%)<br>PGY 3-5: 56 (38.6%)<br>PGY 6-7: 64 (44.1%)<br>Prefer not to say 2 (1.4%)	38.6/60/1.4 (Prefer not to say)	GAD-7[2], Brief-COPE[36]	The COVID-19 pandemic has significant impact on American vascular surgery trainees, which include stress about how the impact will affect their career and anxiety related to a loved becoming infected with COVID-19;<br>They reported health coping mechanisms and had a high level of resiliency and relatively low levels of anxiety
**Kannampallil (2020)(138)**	Cross sectional survey	North America (United States)	COVID-19	393	Physician trainees (fellows and residents)	> = 4 years on the program:<br>No: 311 (80.4%)<br>Yes: 76 (19.6%)	55/45	DASS-21[5], PFI[62],Survey, Items derived from NIOSH Quality of Worklife Questionnair e[63]	Respondents caring for patients currently being tested for COVID-19 had a higher prevalence of stress and burnout. This group (caring for

									patients currently being tested for COVID-19) also experienced moderate to extremely high perceived stress regarding childcare and had lower work family balance. Female trainees and trainees who were exposed to COVID-19 patients reported sig higher stress and were more likely to be burnt out.
**Kelker (2020)(139)**	Prospective cohort study	North America (United States)	COVID-19	157	Emergency medicine	1-5 years: 47 (42)<br>6-10 years: 21 (19)<br>11-15 years: 12 (11)<br>>16 years: 33 (29)	Range % across multiple time points 54-59/36-41/3-4 (Prefer not to say)	PWLS[64], BRS[65], WBI[66], Survey	Concerns for persona safety decreased over 4 weeks from 85% to 61% (p<0.001). Symptoms of stress, fear or anxiety was initially 83% and significantly reduced to 66% (p=0.009). Burnout did not significantly change over 4 weeks.
**Khalafallah (2020)(140) Attendings**	Cross-sectional survey	North America (United States)	COVID-19	407	Neurosurgeons (attending)	<5 (47[11.5%]), 5-15 (91[23.8%]), >15 (263[64.6%])	11.3/88.7	aMBI[33], 5-point Likert scales	Majority of respondents reported a decrease in work hours due to the pandemic (82.6%). Burnout was identified in 83 (20.4%)

									respondents. Rate of burnout was decreased when compared to rates reported pre-COVID
**Khalafallah (2020)(141) Residents**	Cross-sectional survey	North America (United States)	COVID-19	167	Neurosurgery residents	PGY 1: 15 (13.5)<br>PGY 2: 7 (6.3)<br>PGY 3: 19 (17.1)<br>PGY 4: 12 (10.8)<br>PGY 5: 17 (15.3)<br>PGY 6: 23 (20.7)<br>PGY 7: 17 (15.3)<br>PGY >7: 1 (0.9)	33.3/65.8/0.9 (Prefer not to say)	Survey with 3-point Likert items, aMBI[33]	The COVID-19 pandemic impacts neurosurgical residents, with nearly one-third experiencing burnout. Residents who are earlier in their career (i.e., lower post graduate years [PGYs]) experience increased burnout when compared to their more senior colleagues (33.2% reduction in burnout rate with every 1-year increase in PGY level).
**Khusid (2020)(142)**	Cross-sectional survey	North America (United States)	COVID-19	332	Urology residents	Range: 1-5<br><br>Resident year: n (%)<br>PGY1: 53 (16)<br>PGY2: 69 (21)<br>PGY3: 67 (20)<br>PGY4: 56 (17)<br>PGY5: 87 (26)	35.0/65.0	Likert scale questions	Perceived availability of PPE and program support were associated with lower levels of anxiety and depression at work and at home.
**Robbins (2020)(143)**	Cross-sectional survey	North America (United States)	COVID-19	108	Radiology Residents	NR	NR	Survey	Residency programs were negatively impacted by COVID. Residents reported worse morale but reported adequate

									access to mental health resources during the pandemic.
**Rodriguez (2020)(144)**	Cross-sectional survey	North America (United States)	COVID-19	426	Emergency medicine (attending, fellow, resident)	Faculty: 55.4%<br>Fellow: 4.5%<br>Resident: 39.4%	45.1/54.9	Likert scale question	EM physicians experienced substantial workplace and home anxiety during the COVID-19 pandemic. Availability of PPE, COVID-19 testing, and clear communication decrease provider stress.
**Sanghavi (2020)(145)**	Cross-sectional survey	North America (United States)	COVID-19	29	Residents	Range: >1-3 years;<br>(PGY-1: 38%<br>PGY-2: 38%<br>PGY-3: 24%)	62/ 38	Survey, Likert scale, BDI-II[46], PSS-10[4]	Residents reported increased anxiety, stress, fear of spreading COVID-19. Residents reported anxiety regarding the impact on their training and education, and social isolation impacting general well-being.
**Schwartz (2020)(146)**	Cross-sectional survey	North America (United States)	COVID-19	39	Internal medicine residents	NR	NR	Survey	More than one in five resident physicians reported contemplating suicide or self harm during the COVID-19 pandemic
**Shah (2020)(147)**	Cross-sectional survey	North America (United States)	COVID-19	135 (*assuming program directors	Cardiology fellows	NR	21/79	Survey	COVID-19 has impacted interventional cardiology

				are not physicians)					training. Additionally, many fellows felt added stress due to the COVID-19 pandemic.
**Sharma (2020)(148)**	Cross-sectional	North America (United States)	COVID-19	1651	25% were physicians or physicians-in-training<br>ICU director: n = 59 (4%)<br>Attending physician n= 252 (15%)<br>Physician in training, n = 105 (6%)<br><br>PHYSICIAN only specialties:<br>Critical care medicine, 276 (89%)<br>pulmonology, 192 (62%)<br>internal medicine, 47 (15%)<br>neurology, 35 (11%)<br>Physician in training only specialties:<br>Critical care medicine, 77 (73%)<br>pulmonology, 55 (52%)<br>internal medicine, 25 (24%)<br>neurology, 12 (11%)	NR	74/25/0 (Non-binary)/1 (Not disclosed	Survey	HCPs concerns included worries about transmitting to the family/community (66%), emotional distress/burnout (58%), and insufficient PPE (40%). Insufficient PPE access was the strongest predictor of feeling that the hospital is unable to keep providers safe and worries about transmitting infection to families/communities. Addressing poor communication from supervisors, insufficient PPE, and community stigma may improve provider mental wellbeing.
**Shechter (2020)(149)**	Cross-sectional	North America	COVID-19	282	Residents, attendings, fellows	NR	NR	PC-PTSD[67], PHQ-2[1], GAD-2[2], ISI[3]	A higher proportion of RNs experienced

		(United States)							COVID-19-related psychological distress than attending physicians and residents/fellows. There is not a significant difference between physicians and residents/fellows.
**Thomaier (2020)(150)**	Cross-sectional survey	North America (United States)	COVID-19	374	Cancer care physicians	Mean: 12.7 years	NR	PHQ-4[1]	Prevalence of symptoms of anxiety and depression is high in American oncology physicians.
**Guiroy (2020)(151)**	Cross-sectional survey	North/ South America (Latin America)	COVID-19	204	Spine surgeons	0-5 yrs: 16.2%<br>5-10 yrs: 25.4%<br>10-20 yrs: 34.8%<br>>20 yrs: 23.5%	3.4/96.6	PHQ-9[1]	Approximately half of spine surgeons in Latin America report depressive symptoms as measured by the PHQ-9. Overall, 22.1% were deemed to be at high risk of depression
**Foley (2020)(152)**	Cross-sectional	Oceania (Australia, New Zealand)	COVID-19	214<br>Australia: 196 (92%)<br>New Zealand: 18 (8%)	Adult infectious diseases n: 141 (66%)<br>Paediatric infectious diseases n: 21 (10%)<br>Infectious diseases w/ clinical microbiology n: 52 (24%)	Years of experience post-commencement of advanced specialty training, n (%)<br><5 years - N: 45 (21)<br>5–15 years - N: 95 (44)<br>>15 years- N: 74 (35)	NR	Survey	41% (88/214) were worried about becoming infected through occupational exposure.

**Holton (2020)(153)**	Cross-sectional survey	Oceania (Australia)	COVID-19	138	NR	NR	59/39/2 (Other)	Survey, DASS-21[5]	Approximately one quarter of respondents (all HCWs) reported symptoms of psychological distress. Direct contact with people with a COVID-19 diagnoses and being a nurse or midwife were associated with higher anxiety scores. Higher ratings of the health service's pandemic response and staff support strategies were protective against depression, anxiety and stress.
**Khot (2020)(154)**	Qualitative	Oceania (Australia)	COVID-19	12	Obstetrician	NR	NR	Semi-structured interviews	Obstetricians from the state of Victoria reported anxieties about fulfilling roles and responsibilities during the COVID-19 pandemic, with their primary concerns for their family members and patients
**Civantos (2020)(155)**	Cross-sectional survey	South America (Brazil)	COVID-19	163	Head and neck surgeons	NR	25.8/74.2	Mini-Z[60], GAD-7[2], IES-R[6], PHQ-2[1]	Symptoms of burnout (14.7%), anxiety (45.5%), distress (26.3%) and depression (16.0%) were noted by

									physicians during the COVID-19 pandemic, and females, younger physicians and those with previous psychiatric conditions may be experiencing increased symptoms
**Cotrin (2020)(156)**	Cross-sectional survey	South America (Brazil)	COVID-19	179	NR	<5 - >21 years	65.36/34.64	Survey	Physicians reported high levels of fear of being infected with COVID-19 in the workplace and transmitting it to their families. Physicians reported moderate levels of anxiety related to the pandemic, with scores lower than those reported by dentists.
**Malgor (2020)(157)**	Cross-sectional survey	South America (Brazil)	COVID-19	335	Vascular surgeons	<10 years: 173 (38.3)<br>10-20 years: 133 (29.4)<br>>20 years: 146 (32.3)	33.5/66.6	GAD-7[2], Brief-COPE[36]	Brazilian vascular surgeons report low levels of anxiety during the early stages of the pandemic before the surge of infected patients
**Monterrosa-Castro (2020)(158)**	Cross-sectional survey	South America (Colombia)	COVID-19	531	General practitioners	NR	59.5/40.5	GAD-7[2], Work-related Stress Test[68], FCV-19S[47]	The following factors were associated with higher presence of symptoms of anxiety in Colombian general

									practitioners: female, experienced social discrimination, anguish, job disappointment, nightmares and other symptoms of fear related to the COVID-19 pandemic. Lower presence of symptoms of anxiety were associated with feeling protected by state/employer, job satisfaction, and trust in government measures and information.
**Khattab & K. (2020)(159)**	Cross-sectional survey	Various (33 countries)	COVID-19	781	Spine surgeons	≤ 5 yrs: 29.9%<br>5-10 yrs: 20.1%<br>10-15 yrs: 17.9%<br>≥ 15 yrs: 32.1%	NR	K-10[16]	Spine surgeons who worked in university hospitals (vs. private) were affected the least by COVID-19. Availability of PPE was associated with lower psychological stress.
**Sharif (2020)(160)**	Cross-sectional	Various (52 countries)	COVID-19	375	Neurosurgeons	Residents (20.7%)<br>Consultants (79.3%)	NR	SRQ-20[31]	Neurosurgeons experienced feeling unhappy, feeling tense, had insomnia, headaches, and suicidal ideation during the pandemic. Depression was

									higher among physicians who did not receive PPE guidance or considered the workplace unsafe.
**Shalhub (2020)(161)**	Cross-sectional	Various (58 countries - 43.4% from United States; 43.4% from Brazil)	COVID-19	1609 survey responses<br><br>1518 (completed the GAD-7)	Vascular surgeons	In training n:236 (14.7%)<br>In practice <10 years n:549 (34.1%)<br>In practice 10-20 years n:412 (25.6%)<br>In practice >20 years n:412 (25.6%)<br>Most were board-certified vascular surgeons in practice (n = 1328; 82.5%), followed by vascular surgeons in training (n = 236; 14.7%	28.6/70.5	GAD-7[2], Brief COPE[36]	Vascular surgeons globally have been experiencing multiple COVID-19-related stressors, some degree of anxiety was reported by 54.5% of respondents, and 23.3% reported moderate or severe anxiety. ~60% of respondents reported using active coping strategies and "self-distraction", 20% used other avoidant coping strategies. Factors related to decreased anxiety levels were hospital support and the use of positive reframing as an active coping strategy
**An (2020)(162)**	Cross-sectional survey	Various (71 countries)	COVID-19	1124	Frontline	NR	NR	Survey	When asked if centers were taking enough precautionary measures respondents from the UK reported lowest satisfaction

									(22.2%). In contrast, surgeons from China (95.9%) and the Netherlands (78.8%) displayed much higher satisfaction. Connecting this to their fears, when asked about their fear of getting sick or infecting others, participants displayed relatively high scores (mean ± SD of all respondents: 3.7 ± 1.3). Surgeons from Mexico, the U.S and Turkey had the highest scores compared to the Netherlands and China.
**Azoulay (2020)(163)**	Cross-sectional survey	Various (85 countries)	COVID-19	1001	Frontline	NR	34/66	HADS[13], MBI[33]	46.5% of participants showed symptoms of anxiety, 30.2% showed symptoms of depression and 51% showed symptoms of severe burnout. Being female, working in a university-affiliated hospital, living in a city of > 1 million inhabitants were all independently associated with

									anxiety. One of the main independent determinants of depression was female gender while age was independently associated with severe burnout. Clinician's rating of the ethical climate was an independent determinate for depression, anxiety and severe burnout,
**Louie (2020)(164)**	Cross-sectional survey	Various (91 countries)	COVID-19	902	Spine surgeons	<5 years: 161 (25.3)<br>5 to 10 years: 141 (22.2)<br>10 to 15 years: 104 (16.4)<br>15 to 20 years: 117 (18.4)<br>Over 20 years: 113 (17.8)	6.2/93.8	AO Spine COVID-19 and Spine Surgeon Global Impact Survey[69]	Spinal surgeons globally experience similar stressors and worries related to COVID-19. However, there are regional differences that should be considered.
**Sayari (2020)(165)**	Cross-sectional survey	Various (91 countries)	COVID-19	902	Spine surgeons - trauma surgeon, Neurosurgery, Orthopaedics	NR	6.2/93.8	AO Spine COVID-19 and Spine Surgeon Global Impact Survey[69]	Surgeons personal health and comorbidities were found to impact perceptions, delivery of healthcare services and work-related decision making during COVID-19. Clinicians with more than one personal health comorbidities were more likely

									to note their own health as a current stressor.
**Weiner (2020)(166)**	Cross-sectional survey	Various (91 countries)	COVID-19	892	Spine surgeons	All post fellowship; neurosurgery, orthopedics, pediatrics surgery, trauma. 45.4% in academic settings 22.9% privacademics 16.1% private practice 15.6% public hospitals	6.2/93.8	Survey, Likert scale	Respondents from academic institutions reported overall lower levels of worry, and that their centres had adequate access to PPE compared to other practice settings
**Alhaj (2020)(167)**	Cross-sectional survey	Various (Arabia, Canada, Italy, Kuwait, Saudi, Serbia, United States)	COVID-19	52	Neurosurgery Residents	NR	26.9/73.1	Survey, Questionnaire regarding knowledge of COVID-19	The median knowledge score was 4 out of 5 and 60% of participants had a satisfactory level of knowledge regarding COVID-19. The differences in knowledge scores by location were statistically significant. Almost half of the neurosurgery residents interacted with COVID-19 patients and over half of the respondents stated that they had received a session about personal protective

									equipment. About 90% of respondents believed that this pandemic had influenced their mental health.
**Schmulson (2020)(168)**	Cross-Sectional survey	Various (Argentina, Brazil, Colombia, Chile, Ecuador, Guatamala, Mexico, Nicaragua, Panama, Peru, Uruguay)	COVID-19	61	Gastroenterologist (Neurogastroenterology)	NR	42.6/57.4	Survey	All surveyed physicians reported clinical practice and procedures being negatively impacted by COVID-19, as well as reporting experiences of depression and anxiety (78.7% of physicians). Moreover, physicians reported more psychological symptoms if conducting elective endoscoping procedures, in clinical consultations, elective and emergency endoscopic procedures, and if they had a colleague diagnosed with COVID-19.
**Bhargava (2020)(169)**	Cross-sectional survey	Various (Asia, Central/South America, Europe,	COVID-19	733	Dermatologists	less or equal to 10 yrs - over 20	NR	Survey	Most (77.2%) respondents indicated that they experienced some form of mental

		North America, Other)							distress during the pandemic.
**Rajan (2020)(170)**	Cross-sectional survey	Various (Australia, Canada, China, India, Iran, Italy, United Kingdom, Qatar, Spain, United States)	COVID-19	36	Neuroanaesthesiology fellows	NR	NR	Survey (free-text answers)	Neuroanaesthesiology fellows experienced stress due to the postponed of examinations hindering graduation and extension of training. They also experienced anxiety about steering through the pandemic and stress concerning the safety of their family. Adequate breaks from clinical duties and time to communicate with their social support systems may mitigate these psychological impacts
**Kapila (2020)(171)**	Cross-sectional survey	Various (Belgium, Colombia, France, India, Israel, Italy, Romania, Slovakia, The Netherlands, United Kingdom)	COVID-19	86	Plastic surgery residents	Belgium only:<br>3rd year: 9/35 (25.7%)<br>4th year: 8/35 (22.9%)<br>5th year: 9/35 (25.7%)<br>6th year: 9/35 (25.7%)	NR	Questionnaire	Though the COVID-19 pandemic had no significant affect on training of plastic surgery trainees, trainees reported anxiety regarding the health of their loved ones, their own health and the future of their training

**Gokdemir (2020)(172)**	Cross-sectional survey	Various (Canada, Greece, Italy, Portugal, Spain, Turkey, United States, Other)	COVID-19	250	Family Physician	1-43 years	58/41/1 (No report), Gender	Survey, TIPI[70], SWLS[71], PSS[4], DAS[72]	Those with tendency towards conscientiousness, emotional stability, and openness to experience and who were more satisfied with life, and had less death anxiety, reported a more positive attitude towards COVID-19. Those with higher death anxiety reported higher fear of COVID-19. Those who had never caught an infectious disease from a patient reported higher fear of COVID-19 than those who had at least once.
**Ebola**									
**Abebe (2016)(173)**	Cross-sectional survey	Africa (Ethiopia)	Ebola	99	Various (hospital)	NR	NR	Likert scale questions	78.8% of physicians afraid of getting Ebola
**Smith (2017)(174)**	Qualitative	North America (United States)	Ebola	3	Frontline	NR	NR	Categorized interview responses	33% of physicians experienced episodes of discrimination
**Inf. A (H1N1)**									
**El Gaafary (2010)(175)**	Cross-sectional survey	Africa (Egypt)	Inf. A (H1N1)	72	Various (hospital)	NR	44.4/55.6	Likert scale questions	100% of physicians feels their job puts them at increased

									risk of being exposed to H1N1. 93.1% of physicians fear falling ill with H1N1. 91.7% of physicians worry about their family during the H1N1 pandemic. Lower proportion of residents felt ready to face H1N1 (vs. consultants and specialists).
**Austria-Corrales (2011)(176)**	Cross-sectional survey	North America (Mexico)	Inf. A (H1N1)	99	Residents	Median: 2<br>Mean: 2.2<br>Range: 1-5+	44.4/55.6	MBI[33]	36.4% of physicians experience burnout
**Tzeng (2008)(177)**	Cross-sectional survey	Asia (Taiwan)	Inf. A (H5N1)	44	Various (hospital)	NR	NR	Likert scale questions	29.5% of physicians felt fearful about the avian flu epidemic.
**Wong (2008)(178)**	Cross-sectional survey	Asia (Singapore)	Inf. A (H5N1)	285	General practitioners	(range: n (%))<br>1-5: 49 (17.2)<br>6-10: 50 (21.1)<br>11-15: 56 (19.6)<br>16-20: 50 (17.5)<br>>20: 64 (22.5)	36.8/63.2	Likert scale questions	Family physicians fear their job puts them increased risk of exposure to H5N1, are afraid of falling ill with the bird flu, are concerned for their spouse and children, and feared there would be inadequate staff to manage patient demand.
**Tang (2017)(179)**	Cross-sectional survey	Asia (China)	Inf. A (H7N9)	26	ICU, ER, respiratory department (hospital)	NR	NR	TSD Checklist-Civilian	Most physicians experienced stress reactions

								Version (PCL-C)[73]	and eventually developed PTSD
				**MERS**					
**Alsubaie (2019)(180)**	Cross-sectional survey	Asia (Saudi Arabia)	MERS	284	Various (hospital)	Median: 4<br>Range: 1-35	31.3/68.7	Likert scale questions	89.7% of physicians worried about the ability of MERS to cause severe disease or death and experienced anxiety toward the risk of transmitting MERS to their family
				**SARS**					
**Poon (2004)(181)**	Cross-sectional survey	Asia (Hong Kong)	SARS	141	Frontline	Median: 16	31.3/68.7	STAI[23]	Physicians scored a mean anxiety score of 47.8, with higher score among staff who had contact with patients with SARS
**Wong (2004)(182)**	Cross-sectional survey	Asia (Hong Kong)	SARS	137	General practitioners	(range: n (%))<br><10: 22 (16.3)<br>11-20: 54 (40.0)<br>21-30: 41 (30.4)<br>>31: 18 (13.3)	17.6/82.4	Survey	Significant anxiety found in family doctors. Female doctors were more worried about infecting their families, compared to male doctors.
**Wong (2005)(183)**	Cross-sectional survey	Asia (Hong Kong)	SARS	123	Emergency department	NR	NR	Likert scale questions	The mean overall distress level for doctors post-SARS outbreak was 5.91 (where 0 is no distress and 10

									is very distressed) and correlated with vulnerability/loss of control, health of self, spread of virus, health of family, changes in work, and being isolated. Physicians use planning as a coping strategy.
**Chan (2004)(184)**	Cross-sectional survey	Asia (Singapore)	SARS	113	Various (hospital)	NR	NR	GHQ-28[37], IES[6]	As many as 20% of physicians experienced symptoms of PTSD, with single and these psychological symptoms were associated with single marital status.
**Koh (2005)(185)**	Cross-sectional survey	Asia (Singapore)	SARS	NR	Various (hospital)	NR	NR	Likert scale questions	Direct patient contact associated with worse psychological symptoms
**Tan (2006)(186)**	Qualitative	Asia (Singapore)	SARS	8	General practitioners	Mean: 19.6<br>Range: 12-36	25.0/75.0	Categorized interview responses	Family physicians fear for themselves and what would happen to their family and children if they spread the infection to them. Family physicians feel they do not have sufficient training or experience with

									the appropriate use of PPE.
**Tham, 2005(187)**	Cross-sectional survey	Asia (Singapore)	SARS	38	Emergency department	Median: 6 Range: 1-15	34.2/65.8	GHQ-28[37], IES[6]	13.2% of physicians had an IES total score >26, which means they experienced post-event morbidity
**Verma (2004)(188)**	Cross-sectional survey	Asia (Singapore)	SARS	721	General practitioners	NR	38.8/60.6	GHQ-28[37], HSS[74], IES-R[6]	General practitioners directly involved in the care of patients with SARS were significantly more likely to have clinically important psychological distress when compared to general practitioners who did not care for patients with SARS (OR=2.9, 95%CI:1.3-6.3). 37.5% were afraid of infecting self, family, and other loved ones
**Lung (2009)(189)**	Prospective cohort	Asia (Taiwan)	SARS	24	NR	NR	0/100	CHQ[75], General questions	20.8% of physicians experienced psychological symptoms
**Wong (2007)(190)**	Cross-sectional survey	Asia/ North America (Canada, Hong Kong)	SARS	188	General practitioners	69.1% reported over 10 years of experience	23.9/68.6	Likert scale questions	Symptoms of anxiety affected as many as 50% of family physicians

**Grace (2005)(191)**	Cross-sectional survey	North America (Canada)	SARS	193	Academic physicians (hospital)	Mean: 22.2<br>SD: 11.1	32.1/67.9	Likert scale questions	Physicians providing direct care to patients with SARS felt more stigmatized compared with physicians not caring for patients with SARS. Direct physician contact is associated with worse psychological symptoms. 27.5% of physicians worried about SARS spreading to their family.
**Nickell (2004)(192)**	Cross-sectional survey	North America (Canada)	SARS	173	Various (hospital)	NR	NR	GHQ-12[37]	17.4% of physicians experienced emotional distress
**Tolomiczenko (2005)(193)**	Cross-sectional survey	North America (Canada)	SARS	61	Various (hospital)	NR	NR	Likert scale questions	More physicians had fears about coming to the hospital and relied on community-based healthcare workers for support.

Table sorted by outbreak, then alphabetized by continent/country

Abbreviations: MERS, Middle East Respiratory Syndrome; SARS, Severe Acute Respiratory Syndrome; Inf. A, Influenza A virus; COVID-19, Severe acute respiratory virus 2 (SARS-CoV-2); DSM, Diagnostic and Statistical Manual of Mental Disorders (versions indicated by roman numerals IV, IV-TR); HCP, Healthcare Provider; HCW, Healthcare Worker; NIOSH, National Institute of Occupational Health and Safety; PPE, Personal Protective Equipment; NR, Not reported.

References

1. Enyama D, Chelo D, Noukeu Njinkui D, Mayouego Kouam J, Fokam Djike Puepi Y, Mekone Nkwele I, et al. Impact of the COVID-19 pandemic on pediatricians' clinical activity in Cameroon. Archives de Pediatrie. 2020.
2. Ali H, Ismail AA, Abdalwahab A. Mental stress in anesthesia and intensive care physicians during COVID-19 outbreak. Anesthesiology and Pain Medicine. 2020;10(5):1-6.
3. Bakry AMA, Sobhy E, Abdelmohty H. Cardiac surgeons between apprehension and ethical duty in the COVID-19 pandemic. Asian Cardiovascular and Thoracic Annals. 2020.
4. Elkholy H, Tawfik F, Ibrahim I, Salah El-Din W, Sabry M, Mohammed S, et al. Mental health of frontline healthcare workers exposed to COVID-19 in Egypt: A call for action. The International journal of social psychiatry. 2020:20764020960192.
5. Khattab MF, Abou-Madawi AM. Current effect of COVID-19 global pandemic on the professional and life profiles of the Egyptian spine surgeons. Sicotj. 2020;6:31.
6. Elhadi M, Msherghi A. Mental health of surgeons during the COVID-19 pandemic: An urgent need for intervention. Surgery. 2020.
7. Fekih-Romdhane F, Snene H, Jebri A, Ben Rhouma M, Cheour M. Psychological impact of the Pandemic COVID-19 Outbreak Among Medical Residents in Tunisia. Asian Journal of Psychiatry. 2020;53 (no pagination).
8. Halayem S, Sayari N, Cherif W, Cheour M, Damak R. How Tunisian physicians of public health hospitals deal with COVID-19 pandemic: Perceived stress and coping strategies. Psychiatry Clin Neurosci. 2020;74(9):496-7.
9. Arafa A, Mohammed Z, Mahmoud O, Elshazley M, Ewis A. Depressed, anxious, and stressed: What have healthcare workers on the frontlines in Egypt and Saudi Arabia experienced during the COVID-19 pandemic? Journal of Affective Disorders. 2021;278:365-71.
10. Cai H, Tu B, Ma J, Chen L, Fu L, Jiang Y, et al. Psychological Impact and Coping Strategies of Frontline Medical Staff in Hunan Between January and March 2020 During the Outbreak of Coronavirus Disease 2019 (COVID‑19) in Hubei, China. Med Sci Monit. 2020;26:e924171.
11. Huang JZ, Han MF, Luo TD, Ren AK, Zhou XP. [Mental health survey of 230 medical staff in a tertiary infectious disease hospital for COVID-19]. Zhonghua Lao Dong Wei Sheng Zhi Ye Bing Za Zhi/Zhonghua Laodong Weisheng Zhiyebing Zazhi/Chinese Journal of Industrial Hygiene & Occupational Diseases 38(0):E001, 2020 Mar 04. 2020;38(0):E001.
12. Juan Y, Yuanyuan C, Qiuxiang Y, Cong L, Xiaofeng L, Yundong Z, et al. Psychological distress surveillance and related impact analysis of hospital staff during the COVID-19 epidemic in Chongqing, China. Comprehensive Psychiatry. 2020;103 (no pagination).
13. Lai J, Ma S, Wang Y, Cai Z, Hu J, Wei N, et al. Factors Associated With Mental Health Outcomes Among Health Care Workers Exposed to Coronavirus Disease 2019. JAMA Netw Open. 2020;3(3):e203976.
14. Li W, Frank E, Zhao Z, Chen L, Wang Z, Burmeister M, et al. Mental Health of Young Physicians in China During the Novel Coronavirus Disease 2019 Outbreak. JAMA Netw Open. 2020;3(6):e2010705.
15. Li G, Miao J, Wang H, Xu S, Sun W, Fan Y, et al. Psychological impact on women health workers involved in COVID-19 outbreak in Wuhan: A cross-sectional study. Journal of Neurology, Neurosurgery and Psychiatry. 2020;91(8):895-7.
16. Liu Q, Luo D, Haase JE, Guo Q, Wang XQ, Liu S, et al. The experiences of health-care providers during the COVID-19 crisis in China: a qualitative study. Lancet Glob Health. 2020;8(6):e790-e8.
17. Liu Y, Wang L, Chen L, Zhang X, Bao L, Shi Y. Mental Health Status of Paediatric Medical Workers in China During the COVID-19 Outbreak. Frontiers in Psychiatry. 2020;11 (no pagination).
18. Liu Z, Wu J, Shi X, Ma Y, Ma X, Teng Z, et al. Mental Health Status of Healthcare Workers in China for COVID-19 Epidemic. Annals of Global Health. 2020;86(1):128.
19. Ning X, Yu F, Huang Q, Li X, Luo Y, Huang Q, et al. The mental health of neurological doctors and nurses in Hunan Province, China during the initial stages of the COVID-19 outbreak. BMC Psychiatry. 2020;20(1):436.
20. Que J, Shi L, Deng J, Liu J, Zhang L, Wu S, et al. Psychological impact of the COVID-19 pandemic on healthcare workers: a cross-sectional study in China. Gen Psychiatr. 2020;33(3):e100259.
21. Shao Y, Zhang W. Psychological and Ocular Surface State of Ophthalmologists and Ophthalmic Nurses Working With Patients With Coronavirus Disease 2019. JAMA Ophthalmol. 2020;138(8):907-10.
22. Song X, Fu W, Liu X, Luo Z, Wang R, Zhou N, et al. Mental health status of medical staff in emergency departments during the Coronavirus disease 2019 epidemic in China. Brain Behav Immun. 2020;88:60-5.

23. Wang H, Huang D, Huang H, Zhang J, Guo L, Liu Y, et al. The psychological impact of covid-19 pandemic on medical staff in guangdong, china: A cross-sectional study. Psychological Medicine. 2020:No Pagination Specified.
24. Wang Y, Li Y, Jiang J, Feng Y, Lu D, Zhang W, et al. COVID-19 outbreak-related psychological distress among healthcare trainees: a cross-sectional study in China. BMJ Open. 2020;10(10):e041671.
25. Wu J, Chen X, Yao S, Liu R. Anxiety persists after recovery from acquired COVID-19 in anaesthesiologists. J Clin Anesth. 2020;67:109984.
26. Xu Z, Ye Y, Wang Y, Qian Y, Pan J, Lu Y, et al. Primary Care Practitioners' Barriers to and Experience of COVID-19 Epidemic Control in China: a Qualitative Study. Journal of General Internal Medicine. 2020.
27. Yang X, Zhang Y, Li S, Chen X. Risk factors for anxiety of otolaryngology healthcare workers in Hubei province fighting coronavirus disease 2019 (COVID-19). Social psychiatry and psychiatric epidemiology. 2020;12.
28. Zhang Q, Mu MC, He Y, Cai ZL, Li ZC. Burnout in emergency medicine physicians: A meta-analysis and systematic review. Medicine (Baltimore). 2020;99(32):e21462.
29. Zhao X, Zhang T, Li B, Yu X, Ma Z, Cao L, et al. Job-related factors associated with changes in sleep quality among healthcare workers screening for 2019 novel coronavirus infection: a longitudinal study. Sleep Medicine. 2020;75:21-6.
30. Zhu J, Sun L, Zhang L, Wang H, Fan A, Yang B, et al. Prevalence and Influencing Factors of Anxiety and Depression Symptoms in the First-Line Medical Staff Fighting Against COVID-19 in Gansu. Front Psychiatry. 2020;11:386.
31. Bajaj J, Solanki S. Study of risk factors and psychological impact in physicians diagnosed with COVID-19: An online, postexposure, cross-sectional survey. Journal of Anaesthesiology Clinical Pharmacology. 2020;36(3):345-9.
32. Banerjee S, Sarkar S, Bandyopadhyay SN. Survey and analysis of knowledge, attitude and practice among otolaryngologists in a state in eastern India in relation to the coronavirus disease 2019 pandemic. Journal of Laryngology and Otology. 2020;134(8):696-702.
33. Barik S, Paul S, Kandwal P. Insight into the changing patterns in clinical and academic activities of the orthopedic residents during COVID-19 pandemic: a cross-sectional survey. Knee Surgery, Sports Traumatology, Arthroscopy. 2020;28(10):3087-93.
34. Chatterjee SS, Bhattacharyya R, Bhattacharyya S, Gupta S, Das S, Banerjee BB. Attitude, practice, behavior, and mental health impact of COVID-19 on doctors. Indian J Psychiatry. 2020;62(3):257-65.
35. Das A, Sil A, Jaiswal S, Rajeev R, Thole A, Jafferany M, et al. A Study to Evaluate Depression and Perceived Stress Among Frontline Indian Doctors Combating the COVID-19 Pandemic. The primary care companion for CNS disorders. 2020;22(5).
36. Gangakhedkar G, Solanki S. Impact of the COVID-19 pandemic on anesthesiologists in India: A cross-sectional online survey of the practices, preparedness, and mind-set. Journal of Anaesthesiology Clinical Pharmacology. 2020;36(3):331-6.
37. Gupta S, Prasad AS, Dixit PK, Padmakumari P, Gupta S, Abhisheka K. Survey of prevalence of anxiety and depressive symptoms among 1124 healthcare workers during the coronavirus disease 2019 pandemic across India. Medical Journal Armed Forces India. 2020;01:01.
38. Khanna RC, Honavar SG, Metla AL, Bhattacharya A, Maulik PK. Psychological impact of COVID-19 on ophthalmologists-in-training and practising ophthalmologists in India. Indian J Ophthalmol. 2020;68(6):994-8.
39. Khasne RW, Dhakulkar BS, Mahajan HC, Kulkarni AP. Burnout among healthcare workers during COVID-19 pandemic in india: Results of a questionnaire-based survey. Indian Journal of Critical Care Medicine. 2020;24(8):664-71.
40. Mishra D, Nair AG, Gandhi RA, Gogate PJ, Mathur S, Bhushan P, et al. The impact of COVID-19 related lockdown on ophthalmology training programs in India - Outcomes of a survey. Indian J Ophthalmol. 2020;68(6):999-1004.
41. Pandey U, Corbett G, Mohan S, Reagu S, Kumar S, Farrell T, et al. Anxiety, Depression and Behavioural Changes in Junior Doctors and Medical Students Associated with the Coronavirus Pandemic: A Cross-Sectional Survey. Journal of Obstetrics and Gynecology of India. 2020.
42. Podder I, Agarwal K, Datta S. Comparative analysis of perceived stress in dermatologists and other physicians during national lock-down and COVID-19 pandemic with exploration of possible risk factors: A web-based cross-sectional study from Eastern India. Dermatol Ther. 2020;33(4):e13788.
43. Sahu D, Agrawal T, Rathod V, Bagaria V. Impact of COVID 19 lockdown on orthopaedic surgeons in India: A survey. J Clin Orthop Trauma. 2020;11(Suppl 3):S283-s90.
44. Sarma R, Vig S, Rathore P, Pushpam D, Mishra S, Gupta N, et al. Concerns of Health Care Professionals Managing non-COVID Patients during The COVID-19 Pandemic: A Descriptive Cross- Sectional Study. Indian Journal of Palliative Care. 2020;26(Suppl 1):S21-S6.
45. Saurabh K, Ranjan S. Preparedness, perceived impact and concerns of health care workers in a teaching hospital during coronavirus disease 2019 (COVID-19). Journal of Family Medicine & Primary Care. 2020;9(8):4247-51.

46. Sil A, Das A, Jaiswal S, Jafferany M, Thole A, Rajeev R, et al. Mental health assessment of frontline COVID-19 dermatologists: A Pan-Indian multicentric cross-sectional study. Dermatol Ther. 2020:e13884.
47. Singariya G, Kamal M, Kumar M, Jain A, Solanki RK. COVID-19 pandemic: Psychological impact on anaesthesiologists. Indian Journal of Anaesthesia. 2020;64(9):774-83.
48. Suryavanshi N, Kadam A, Dhumal G, Nimkar S, Mave V, Gupta A, et al. Mental health and quality of life among healthcare professionals during the COVID-19 pandemic in India. Brain and Behavior. 2020.
49. Uvais NA, Shihabudheen P, Hafi NAB. Perceived Stress and Stigma Among Doctors Working in COVID-19-Designated Hospitals in India. The primary care companion for CNS disorders. 2020;22(4).
50. Naser Moghadasi A. Evaluation of the Level of Anxiety among Iranian Multiple Sclerosis Fellowships During the Outbreak of COVID-19. Arch Iran Med. 2020;23(4):283.
51. Vafaei H, Roozmeh S, Hessami K, Kasraeian M, Asadi N, Faraji A, et al. Obstetrics Healthcare Providers' Mental Health and Quality of Life During COVID-19 Pandemic: Multicenter Study from Eight Cities in Iran. Psychology Research & Behavior Management. 2020;13:563-71.
52. Abdulah DM, Mohammed AA. The consequences of the COVID-19 pandemic on perceived stress in clinical practice: Experience of Doctors in Iraqi Kurdistan. Rom j intern med. 2020;03.
53. Abdulah DM, Musa DH. Insomnia and stress of physicians during COVID-19 outbreak. Sleep Medicine: X. 2020;2 (no pagination).
54. Milgrom Y, Richter V. Stress assessment among internal medicine residents in a level-3 hospital versus a level-2 hospital with only emergency room service for COVID-19. Journal of Community Hospital Internal Medicine Perspectives. 2020;10(4):301-5.
55. Milgrom Y, Tal Y, Finestone AS. Comparison of hospital worker anxiety in COVID-19 treating and non-treating hospitals in the same city during the COVID-19 pandemic. Israel Journal of Health Policy Research. 2020;9(1).
56. Mosheva M, Hertz-Palmor N, Dorman Ilan S, Matalon N, Pessach IM, Afek A, et al. Anxiety, pandemic-related stress and resilience among physicians during the COVID-19 pandemic. Depression and Anxiety. 2020;37(10):965-71.
57. Awano N, Oyama N, Akiyama K, Inomata M, Kuse N, Tone M, et al. Anxiety, Depression, and Resilience of Healthcare Workers in Japan During the Coronavirus Disease 2019 Outbreak. Internal medicine (Tokyo, Japan). 2020;59(21):2693-9.
58. Naser AY, Dahmash EZ, Al-Rousan R, Alwafi H, Alrawashdeh HM, Ghoul I, et al. Mental health status of the general population, healthcare professionals, and university students during 2019 coronavirus disease outbreak in Jordan: a cross-sectional study. medRxiv. 2020.
59. Saadeh RA, Alfaqih M, Younis OB, Okour A, Obeidat K. The psychosocial and clinical concerns of physicians treating COVID-19 patients. Journal of Taibah University Medical Sciences. 2020;23:23.
60. Mohd Fauzi MF, Mohd Yusoff H, Muhamad Robat R, Mat Saruan NA, Ismail KI, Mohd Haris AF. Doctors' Mental Health in the Midst of COVID-19 Pandemic: The Roles of Work Demands and Recovery Experiences. International Journal of Environmental Research & Public Health [Electronic Resource]. 2020;17(19):08.
61. Amin F, Sharif S, Saeed R, Durrani N, Jilani D. COVID-19 pandemic- knowledge, perception, anxiety and depression among frontline doctors of Pakistan. BMC Psychiatry. 2020;20(1).
62. Arshad AR, Islam F. COVID-19 and Anxiety amongst Doctors: A Pakistani Perspective. Journal of the College of Physicians and Surgeons--Pakistan : JCPSP. 2020;30(10):106-9.
63. Ayub M, Arshad D, Maqbool N, Zahid M, Malik RS, Rizvi ZA, et al. Physicians' Attitudes Towards Treating Patients in the Context of COVID-19 Pandemic in Pakistan. Cureus. 2020;12(9):e10331.
64. Hasan SR, Hamid Z, Jawaid MT, Ali RK. Anxiety among doctors during COVID-19 pandemic in secondary and tertiary care hospitals. Pakistan Journal of Medical Sciences. 2020;36(6):1360-5.
65. Imran N, Masood HMU, Ayub M, Gondal KM. Psychological impact of COVID-19 pandemic on postgraduate trainees: a cross-sectional survey. Postgraduate Medical Journal. 2020;25.
66. Osama M, Zaheer F, Saeed H, Anees K, Jawed Q, Syed SH, et al. Impact of COVID-19 on surgical residency programs in Pakistan; A residents' perspective. Do programs need formal restructuring to adjust with the "new normal"? A cross-sectional survey study. International Journal Of Surgery. 2020;79:252-6.
67. Rashid A, Faisal K. Pandemic anxiety and its correlates among young doctors working frontline in Pakistan. Global Mental Health. 2020;(no pagination).
68. Urooj U, Ansari A, Siraj A, Khan S, Tariq H. Expectations, Fears and Perceptions of doctors during Covid-19 Pandemic. Pak J Med Sci. 2020;36(Covid19-s4):S37-s42.
69. Sorokin MY, Kasyanov ED, Rukavishnikov GV, Makarevich OV, Neznanov NG, Morozov PV, et al. Stress and stigmatization in health-care workers during the COVID-19 pandemic. Indian Journal of Psychiatry. 2020;62(9 Supplement 3):S445-S53.

70. Al Sulais E, Mosli M, AlAmeel T. The psychological impact of COVID-19 pandemic on physicians in Saudi Arabia: A cross-sectional study. Saudi J Gastroenterol. 2020;26(5):249-55.
71. Alahmadi AS, Alhatlan HM, Bin Helayel H, Khandekar R, Al Habash A, Al-Shahwan S. Residents' Perceived Impact of COVID-19 on Saudi Ophthalmology Training Programs-A Survey. Clinical Ophthalmology. 2020;14:3755-61.
72. AlAteeq DA, Aljhani S, Althiyabi I, Majzoub S. Mental health among healthcare providers during coronavirus disease (COVID-19) outbreak in Saudi Arabia. Journal of Infection and Public Health. 2020;13(10):1432-7.
73. Almater A, Tobaigy M, Younis A, Alaqeel M, Abouammoh M. Effect of 2019 coronavirus pandemic on ophthalmologists practicing in Saudi Arabia: A psychological health assessment. Middle East African Journal of Ophthalmology. 2020;27(2):79-85.
74. Alnofaiey YH, Alshehri HA, Alosaimi MM, Alswat SH, Alswat RH, Alhulayfi RM, et al. Sleep disturbances among physicians during COVID-19 pandemic. BMC Research Notes. 2020;13(1):493.
75. Chew QH, Chia FLA, Ng WK, Lee WCI, Tan PLL, Wong CS, et al. Psychological and coping responses to COVID-19 amongst residents in training across ACGME-I accredited specialties in Singapore. Psychiatry Research. 2020;290 (no pagination).
76. Lee MCC, Thampi S, Chan HP, Khoo D, Chin BZB, Foo DPX, et al. Psychological distress during the COVID-19 pandemic amongst anaesthesiologists and nurses. British Journal of Anaesthesia. 2020;125(4):e384-e6.
77. Tan YQ, Chan MT, Chiong E. Psychological health among surgical providers during the COVID-19 pandemic: a call to action. British Journal of Surgery. 2020;107(11):e459-e60.
78. Chang DG, Park JB, Baek GH, Kim HJ, Bosco A, Hey HWD, et al. The impact of COVID-19 pandemic on orthopaedic resident education: a nationwide survey study in South Korea. International Orthopaedics. 2020;44(11):2203-10.
79. Jo SH, Koo BH, Seo WS, Yun SH, Kim HG. The psychological impact of the coronavirus disease pandemic on hospital workers in Daegu, South Korea. Comprehensive Psychiatry. 2020;103 (no pagination).
80. Al Mahyijari N, Badahdah AM, Khamis F. The Psychological Impacts of COVID-19: A Study of Frontline Physicians and Nurses in the Arab World. Irish Journal of Psychological Medicine. 2020.
81. Kuo FL, Yang PH, Hsu HT, Su CY, Chen CH, Yeh IJ, et al. Survey on perceived work stress and its influencing factors among hospital staff during the COVID-19 pandemic in Taiwan. Kaohsiung Journal of Medical Sciences. 2020.
82. Caliskan F, Dost B. The evaluation of knowledge, attitudes, depression and anxiety levels among emergency physicians during the COVID-19 pandemic. Signa Vitae. 2020;16(1):163-71.
83. Elbay RY, Kurtulmuş A, Arpacıoğlu S, Karadere E. Depression, anxiety, stress levels of physicians and associated factors in Covid-19 pandemics. Psychiatry Res. 2020;290:113130.
84. Hacimusalar Y, Kahve AC, Yasar AB, Aydin MS. Anxiety and hopelessness levels in COVID-19 pandemic: A comparative study of healthcare professionals and other community sample in Turkey. Journal of Psychiatric Research. 2020;129:181-8.
85. Korkmaz S, Kazgan A, Cekic S, Tartar AS, Balci HN, Atmaca M. The anxiety levels, quality of sleep and life and problem-solving skills in healthcare workers employed in COVID-19 services. Journal of Clinical Neuroscience. 2020;80:131-6.
86. Kurt O, Deveci SE, Oguzoncul AF. Levels of anxiety and depression related to covid-19 among physicians: An online cross-sectional study from turkey. Annals of Clinical and Analytical Medicine. 2020;11(Supplement 3):S288-S93.
87. Sahin MK, Aker S, Sahin G, Karabekiroglu A. Prevalence of Depression, Anxiety, Distress and Insomnia and Related Factors in Healthcare Workers During COVID-19 Pandemic in Turkey. Journal of community health. 2020;45(6):1168-77.
88. Saracoglu KT, Simsek T, Kahraman S, Bombaci E, Sezen O, Saracoglu A, et al. The Psychological Impact of COVID-19 Disease is more Severe on Intensive Care Unit Healthcare Providers: A Cross-sectional Study. Clinical Psychopharmacology & Neuroscience. 2020;18(4):607-15.
89. TaŞ BG, Özceylan G, Öztürk GZ, Toprak D. Evaluation of Job Strain of Family Physicians in COVID-19 Pandemic Period- An Example from Turkey. J Community Health. 2020:1-9.
90. Uyaroglu OA, Basaran NC, Ozisik L, Karahan S, Tanriover MD, Guven GS, et al. Evaluation of the effect of COVID-19 pandemic on anxiety severity of physicians working in the internal medicine department of a tertiary care hospital: a cross-sectional survey. Internal Medicine Journal. 2020.
91. Yalçın Bahat P, Aldıkaçtıoğlu Talmaç M, Bestel A, Topbas Selcuki NF, Karadeniz O, Polat I. Evaluating the effects of the COVID-19 pandemic on the physical and mental well-being of obstetricians and gynecologists in Turkey. Int J Gynaecol Obstet. 2020;151(1):67-73.

92. O'Kelly F, Sparks S, Seideman C, Gargollo P, Granberg C, Ko J, et al. A survey and panel discussion of the effects of the COVID-19 pandemic on paediatric urological productivity, guideline adherence and provider stress. Journal of Pediatric Urology. 2020;16(4):492.e1-.e9.
93. Degraeve A, Lejeune S, Muilwijk T, Poelaert F, Piraprez M, Svistakov I, et al. When residents work less, they feel better: Lessons learned from an unprecedent context of lockdown. Progres en urologie : journal de l'Association francaise d'urologie et de la Societe francaise d'urologie. 2020;08.
94. Salopek-Ziha D, Hlavati M, Gvozdanovi Z, Gasi M, Placento H, Jaki H, et al. Differences in distress and coping with the covid-19 stressor in nurses and physicians. Psychiatria Danubina. 2020;32(2):287-93.
95. Abdessater M, Rouprêt M, Misrai V, Matillon X, Gondran-Tellier B, Freton L, et al. COVID19 pandemic impacts on anxiety of French urologist in training: Outcomes from a national survey. Prog Urol. 2020;30(8-9):448-55.
96. Florin M, Pinar U, Chavigny E, Bouaboula M, Jarboui L, Coulibaly A, et al. Socio-economic and psychological impact of the COVID-19 outbreak on private practice and public hospital radiologists. European Journal of Radiology. 2020;132 (no pagination).
97. Hilmi M, Boileve A, Ducousso A, Michalet M, Turpin A, Neuzillet C, et al. Professional and Psychological Impacts of the COVID-19 Pandemic on Oncology Residents: A National Survey. JCO global oncology. 2020;6:1674-83.
98. Vallee M, Kutchukian S, Pradere B, Verdier E, Durbant E, Ramlugun D, et al. Prospective and observational study of COVID-19's impact on mental health and training of young surgeons in France. British Journal of Surgery. 2020;107(11):e486-e8.
99. Bohlken J, Schömig F, Seehagen T, Köhler S, Gehring K, Roth-Sackenheim C, et al. [Experience of Practice-Based Psychiatrists and Neurologists During the COVID-19 Pandemic]. Psychiatr Prax. 2020;47(4):214-7.
100. Büntzel J, Klein M, Keinki C, Walter S, Büntzel J, Hübner J. Oncology services in corona times: a flash interview among German cancer patients and their physicians. J Cancer Res Clin Oncol. 2020;146(10):2713-5.
101. Kramer V, Papazova I, Thoma A, Kunz M, Falkai P, Schneider-Axmann T, et al. Subjective burden and perspectives of German healthcare workers during the COVID-19 pandemic. European Archives of Psychiatry and Clinical Neuroscience. 2020.
102. Skoda EM, Teufel M, Stang A, Jockel KH, Junne F, Weismuller B, et al. Psychological burden of healthcare professionals in Germany during the acute phase of the COVID-19 pandemic: differences and similarities in the international context. Journal of Public Health. 2020;07.
103. Zerbini G, Ebigbo A, Reicherts P, Kunz M, Messman H. Psychosocial burden of healthcare professionals in times of COVID-19 - a survey conducted at the University Hospital Augsburg. Ger Med Sci. 2020;18:Doc05.
104. Diomidous M. Sleep and Motion Disorders of Physicians and Nurses Working in Hospitals Facing the Pandemic of COVID 19. Medical archives (Sarajevo, Bosnia and Herzegovina). 2020;74(3):210-5.
105. Farooq AR, Iqbal S, Abdul Aziz N, Amasayb A, O'Brien TN, Maher E, et al. COVID-19 pandemic: Impact on doctors in training. Annals of Oncology. 2020;31 (Supplement 4):S1012.
106. Amerio A, Bianchi D, Santi F, Costantini L, Odone A, Signorelli C, et al. Covid-19 pandemic impact on mental health: a web-based cross-sectional survey on a sample of Italian general practitioners. Acta Biomed. 2020;91(2):83-8.
107. Bitonti G, Palumbo AR, Gallo C, Rania E, Saccone G, De Vivo V, et al. Being an obstetrics and gynaecology resident during the COVID-19: Impact of the pandemic on the residency training program. European Journal of Obstetrics, Gynecology, & Reproductive Biology. 2020;253:48-51.
108. Buselli R, Corsi M, Baldanzi S, Chiumiento M, Del Lupo E, Dell'Oste V, et al. Professional Quality of Life and Mental Health Outcomes among Health Care Workers Exposed to Sars-Cov-2 (Covid-19). International Journal of Environmental Research & Public Health [Electronic Resource]. 2020;17(17):26.
109. Sio SD, Buomprisco G, Torre GL, Lapteva E, Perri R, Greco E, et al. The impact of COVID-19 on doctors' well-being: results of a web survey during the lockdown in Italy. European Review for Medical and Pharmacological Sciences. 2020;24(14):7869-79.
110. Di Monte C, Monaco S, Mariani R, Di Trani M. From Resilience to Burnout: Psychological Features of Italian General Practitioners During COVID-19 Emergency. Frontiers in Psychology. 2020;11:567201.
111. Vanni G, Materazzo M, Santori F, Pellicciaro M, Costesta M, Orsaria P, et al. The Effect of coronavirus (COVID-19) on breast cancer teamwork: A multicentric survey. In Vivo. 2020;34:1685-94.
112. Gallopeni F, Bajraktari I, Selmani E, Tahirbegolli IA, Sahiti G, Muastafa A, et al. Anxiety and depressive symptoms among healthcare professionals during the Covid-19 pandemic in Kosovo: A cross sectional study. Journal of Psychosomatic Research. 2020;137 (no pagination).
113. Johnson SU, Ebrahimi OV, Hoffart A. PTSD symptoms among health workers and public service providers during the COVID-19 outbreak. PLoS One. 2020;15(10 October).

114. Rajwa P, Przydacz M, Zapala P, Wieckiewicz G, Ryszawy J, Choragwicki D, et al. How has the COVID-19 pandemic impacted Polish urologists? Results from a national survey. Central European Journal of Urology. 2020;73(3):252-9.
115. Rymarowicz J, Stefura T, Major P, Szeliga J, Wallner G, Nowakowski M, et al. General surgeons' attitudes towards COVID-19: A national survey during the SARS-CoV-2 virus outbreak. European Surgery Acta Chirurgica Austriaca. 2020.
116. Dimitriu MCT, Pantea-Stoian A, Smaranda AC, Nica AA, Carap AC, Constantin VD, et al. Burnout syndrome in Romanian medical residents in time of the COVID-19 pandemic. Med Hypotheses. 2020;144:109972.
117. Guillen-Astete C, Penedo-Alonso R, Gallego-Rodriguez P, Carballo-Cardona C, Estevez-Rueda MJ, Galli-Cambiaso E, et al. Levels of anxiety and depression among emergency physicians in madrid during the sars-cov-2 pandemic. [Spanish]. Emergencias. 2020;32(5):369-73.
118. Martinez-Lopez JA, Lazaro-Perez C, Gomez-Galan J, Fernandez-Martinez MM. Psychological impact of covid-19 emergency on health professionals: Burnout incidence at the most critical period in spain. Journal of Clinical Medicine. 2020;9(9):1-18.
119. Ruiz-Fernandez MD, Ramos-Pichardo JD, Ibanez-Masero O, Cabrera-Troya J, Carmona-Rega MI, Ortega-Galan AM. Compassion fatigue, burnout, compassion satisfaction, and perceived stress in healthcare professionals during the COVID-19 health crisis in Spain. Journal of clinical nursing. 2020;28.
120. Coyle C, Ghazi H, Georgiou I. The mental health and well-being benefits of exercise during the COVID-19 pandemic: a cross-sectional study of medical students and newly qualified doctors in the UK. Irish Journal of Medical Science. 2020.
121. Faderani R, Monks M, Peprah D, Colori A, Allen L, Amphlett A, et al. Improving wellbeing among UK doctors redeployed during the COVID-19 pandemic. Future Healthcare Journal. 2020;7(3):e71-e6.
122. Norton EJ, Georgiou I, Fung A, Nazari A, Bandyopadhyay S, Saunders KEA. Personal protective equipment and infection prevention and control: a national survey of UK medical students and interim foundation doctors during the COVID-19 pandemic. Journal of Public Health. 2020;23.
123. Payne A, Rahman R, Bullingham R, Vamadeva S, Alfa-Wali M. Redeployment of Surgical Trainees to Intensive Care During the COVID-19 Pandemic: Evaluation of the Impact on Training and Wellbeing. Journal of Surgical Education. 2020.
124. Rimmer A. Covid-19: Two fifths of doctors say pandemic has worsened their mental health. BMJ (Clinical research ed). 2020;371:m4148.
125. Shah N, Raheem A, Sideris M, Velauthar L, Saeed F. Mental health amongst obstetrics and gynaecology doctors during the COVID-19 pandemic: Results of a UK-wide study. European Journal of Obstetrics and Gynecology and Reproductive Biology. 2020;253:90-4.
126. de Wit K, Mercuri M, Wallner C, Clayton N, Archambault P, Ritchie K, et al. Canadian emergency physician psychological distress and burnout during the first 10 weeks of COVID-19: A mixed-methods study. Journal of the American College of Emergency Physicians open. 2020;26:26.
127. Gill S, Hao D, Hirte H, Campbell A, Colwell B. Impact of COVID-19 on Canadian medical oncologists and cancer care: Canadian Association of Medical Oncologists survey report. Curr Oncol. 2020;27(2):71-4.
128. Odedra D, Chahal BS, Patlas MN. Impact of COVID-19 on Canadian Radiology Residency Training Programs. Can Assoc Radiol J. 2020;71(4):482-9.
129. Pilar A, Gravel SB, Croke J, Soliman H, Chung P, Wong RKS. Coronavirus Disease 2019's (COVID-19's) Silver Lining-Through the Eyes of Radiation Oncology Fellows. Advances in radiation oncology. 2020;23:23.
130. Civantos AM, Byrnes Y, Chang C, Prasad A, Chorath K, Poonia SK, et al. Mental health among otolaryngology resident and attending physicians during the COVID-19 pandemic: National study. Head Neck. 2020;42(7):1597-609.
131. Collins C, Mahuron K, Bongiovanni T, Lancaster E, Sosa JA, Wick E. Stress and the Surgical Resident in the COVID-19 Pandemic. Journal of Surgical Education. 2020;25:25.
132. Demirjian NL, Fields BKK, Song C, Reddy S, Desai B, Cen SY, et al. Impacts of the Coronavirus Disease 2019 (COVID-19) pandemic on healthcare workers: A nationwide survey of United States radiologists. Clinical Imaging. 2020;68:218-25.
133. Fargen KM, Leslie-Mazwi TM, Klucznik RP, Wolfe SQ, Brown P, Ansari SA, et al. The professional and personal impact of the coronavirus pandemic on US neurointerventional practices: A nationwide survey. Journal of NeuroInterventional Surgery. 2020;12(10):927-31.
134. Fitzpatrick K, Patterson R, Morley K, Stoltzfus J, Stankewicz H. Physician Wellness During a Pandemic. The western journal of emergency medicine. 2020;24.
135. Huffman EM, Athanasiadis DI, Anton NE, Haskett LA, Doster DL, Stefanidis D, et al. How resilient is your team? Exploring healthcare providers' well-being during the COVID-19 pandemic. American Journal of Surgery. 2020.
136. Jha S, Shah S, Calderon MD, Soin A, Manchikanti L. The effect of covid-19 on interventional pain management practices: A physician burnout survey. Pain Physician. 2020;23(4 Special Issue):S271-S82.
137. Johnson AP, Wohlauer MV, Mouawad NJ, Malgor RD, Coogan SM, Sheahan MG, 3rd, et al. The impact of the COVID-19 pandemic on vascular surgery trainees in the United States. Annals of Vascular Surgery. 2020;03:03.

138. Kannampallil TG, Goss CW, Evanoff BA, Strickland JR, McAlister RP, Duncan J. Exposure to COVID-19 patients increases physician trainee stress and burnout. PLoS One. 2020;15(8 August).
139. Kelker H, Yoder K, Musey P, Harris M, Johnson O, Sarmiento E, et al. Longitudinal Prospective Study of Emergency Medicine Provider Wellness Across Ten Academic and Community Hospitals During the Initial Surge of the COVID-19 Pandemic. Research Square. 2020;15:15.
140. Khalafallah AM, Lam S, Gami A, Dornbos DL, Sivakumar W, Johnson JN, et al. Burnout and career satisfaction among attending neurosurgeons during the COVID-19 pandemic. Clinical Neurology and Neurosurgery. 2020;198 (no pagination).
141. Khalafallah AM, Lam S, Gami A, Dornbos DL, Sivakumar W, Johnson JN, et al. A national survey on the impact of the COVID-19 pandemic upon burnout and career satisfaction among neurosurgery residents. Journal of Clinical Neuroscience. 2020;80:137-42.
142. Khusid JA, Weinstein CS, Becerra AZ, Kashani M, Robins DJ, Fink LE, et al. Well-being and education of urology residents during the COVID-19 pandemic: Results of an American National Survey. Int J Clin Pract. 2020;74(9):e13559.
143. Robbins JB, England E, Patel MD, DeBenedectis CM, Sarkany DS, Heitkamp DE, et al. COVID-19 Impact on Well-Being and Education in Radiology Residencies: A Survey of the Association of Program Directors in Radiology. Academic Radiology. 2020;27(8):1162-72.
144. Rodriguez RM, Medak AJ, Baumann BM, Lim S, Chinnock B, Frazier R, et al. Academic Emergency Medicine Physicians' Anxiety Levels, Stressors, and Potential Stress Mitigation Measures During the Acceleration Phase of the COVID-19 Pandemic. Acad Emerg Med. 2020;27(8):700-7.
145. Sanghavi PB, Au Yeung K, Sosa CE, Veesenmeyer AF, Limon JA, Vijayan V. Effect of the Coronavirus Disease 2019 (COVID-19) Pandemic on Pediatric Resident Well-Being. Journal of Medical Education & Curricular Development. 2020;7:2382120520947062.
146. Schwartz DA, Connerney MA, Davila-Molina M, Tummalapalli SL. Resident Mental Health at the Epicenter of the COVID-19 Pandemic. Academic Medicine. 2020;25:25.
147. Shah S, Castro-Dominguez Y, Gupta T, Attaran R, Byrum GV, 3rd, Taleb A, et al. Impact of the COVID-19 pandemic on interventional cardiology training in the United States. Catheterization & Cardiovascular Interventions. 2020;96(5):997-1005.
148. Sharma M, Creutzfeldt CJ, Lewis A, Patel PV, Hartog C, Jannotta GE, et al. Healthcare professionals' perceptions of critical care resource availability and factors associated with mental well-being during COVID-19: Results from a US survey. Clinical infectious diseases : an official publication of the Infectious Diseases Society of America. 2020;02.
149. Shechter A, Diaz F, Moise N, Anstey DE, Ye S, Agarwal S, et al. Psychological distress, coping behaviors, and preferences for support among New York healthcare workers during the COVID-19 pandemic. Gen Hosp Psychiatry. 2020;66:1-8.
150. Thomaier L, Teoh D, Jewett P, Beckwith H, Parsons H, Yuan J, et al. Emotional health concerns of oncology physicians in the United States: fallout during the COVID-19 pandemic. PLoS One. 2020;15(11):e0242767.
151. Guiroy A, Gagliardi M, Coombes N, Landriel F, Zanardi C, Camino Willhuber G, et al. COVID-19 Impact Among Spine Surgeons in Latin America. Global Spine J. 2020:2192568220928032.
152. Foley DA, Chew R, Raby E, Tong SYC, Davis JS, Australasian Society for Infectious Diseases Clinical Research N. COVID-19 in the pre-pandemic period: a survey of the time commitment and perceptions of infectious diseases physicians in Australia and New Zealand. Internal Medicine Journal. 2020;50(8):924-30.
153. Holton S, Wynter K, Trueman M, Bruce S, Sweeney S, Crowe S, et al. Psychological well-being of Australian hospital clinical staff during the COVID-19 pandemic. Australian health review : a publication of the Australian Hospital Association. 2020;09.
154. Khot N, Kumar A. Flattening the anxiety curve: Obstetricians' response to the COVID-19 pandemic in Victoria. Australian and New Zealand Journal of Obstetrics and Gynaecology. 2020;60(4):E10.
155. Civantos AM, Bertelli A, Goncalves A, Getzen E, Chang C, Long Q, et al. Mental health among head and neck surgeons in Brazil during the COVID-19 pandemic: A national study. American Journal of Otolaryngology - Head and Neck Medicine and Surgery. 2020;41(6).
156. Cotrin P, Moura W, Gambardela-Tkacz CM, Pelloso FC, Santos LD, Carvalho MDB, et al. Healthcare Workers in Brazil during the COVID-19 Pandemic: A Cross-Sectional Online Survey. Inquiry : a journal of medical care organization, provision and financing. 2020;57:46958020963711.
157. Malgor RD, Sobreira ML, Mouawad NJ, Johnson AP, Wohlauer MV, Coogan SM, et al. Brazilian vascular surgeons experience during the coronavirus (COVID-19) pandemic. Vascular. 2020.
158. Monterrosa-Castro A, Redondo-Mendoza V, Mercado-Lara M. Psychosocial factors associated with symptoms of generalized anxiety disorder in general practitioners during the COVID-19 pandemic. Journal of Investigative Medicine. 2020;68(7):1228-34.
159. Khattab MF, Kannan TMA, Morsi A, Al-Sabbagh Q, Hadidi F, Al-Sabbagh MQ, et al. The short-term impact of COVID-19 pandemic on spine surgeons: a cross-sectional global study. Eur Spine J. 2020;29(8):1806-12.

160. Sharif S, Amin F, Hafiz M, Benzel E, Peev N, Dahlan RH, et al. COVID 19-Depression and Neurosurgeons. World Neurosurg. 2020;140:e401-e10.
161. Shalhub S, Mouawad NJ, Malgor RD, Johnson AP, Wohlauer MV, Coogan SM, et al. Global vascular surgeons' experience, stressors, and coping during the coronavirus disease 2019 pandemic. Journal of Vascular Surgery. 2020.
162. An Y, Bellato V, Konishi T, Pellino G, Sica GS, Kefleyesus A, et al. Surgeons' fear of getting infected by COVID19: A global survey. British Journal of Surgery. 2020;107(11):e543-e4.
163. Azoulay E, De Waele J, Ferrer R, Staudinger T, Borkowska M, Povoa P, et al. Symptoms of burnout in intensive care unit specialists facing the COVID-19 outbreak. Annals of Intensive Care. 2020;10(1).
164. Louie PK, Harada GK, McCarthy MH, Germscheid N, Cheung JPY, Neva MH, et al. The Impact of COVID-19 Pandemic on Spine Surgeons Worldwide. Global Spine Journal. 2020;10(5):534-52.
165. Sayari AJ, Harada GK, Louie PK, McCarthy MH, Nolte MT, Mallow GM, et al. Personal health of spine surgeons can impact perceptions, decision-making and healthcare delivery during the COVID-19 pandemic-a worldwide study. Neurospine. 2020;17(2):313-30.
166. Weiner JA, Swiatek PR, Johnson DJ, Louie PK, Harada GK, McCarthy MH, et al. Spine Surgery and COVID-19: The Influence of Practice Type on Preparedness, Response, and Economic Impact. Global Spine Journal. 2020.
167. Alhaj AK, Al-Saadi T, Mohammad F, Alabri S. Neurosurgery Residents' Perspective on COVID-19: Knowledge, Readiness, and Impact of this Pandemic. World Neurosurgery. 2020;139:e848-e58.
168. Schmulson M, Gudino-Zayas M, Hani A. The Impact of COVID-19 Pandemic on Neurogastroenterologists in Latin America: Results of an Online Survey. Journal of Clinical Gastroenterology. 2020.
169. Bhargava S, Sarkar R, Kroumpouzos G. Mental distress in dermatologists during COVID-19 pandemic: Assessment and risk factors in a global, cross-sectional study. Dermatologic Therapy. 2020.
170. Rajan S, Bebawy J, Avitsian R, Lee CZ, Rath G, Luoma A, et al. The Impact of the Global SARS-CoV-2 (COVID-19) Pandemic on Neuroanesthesiology Fellowship Programs Worldwide and the Potential Future Role for ICPNT Accreditation. Journal of Neurosurgical Anesthesiology. 2020;16:16.
171. Kapila AK, Schettino M, Farid Y, Ortiz S, Hamdi M. The Impact of Coronavirus Disease 2019 on Plastic Surgery Training: The Resident Perspective. Plastic and Reconstructive Surgery - Global Open. 2020;8(7):e3054.
172. Gokdemir O, Pak H, Bakola M, Bhattacharya S, Hoedebecke K, Jelastopulu E. Family Physicians' Knowledge about and Attitudes towards COVID-19 - A Cross-sectional Multicentric Study. Infection & Chemotherapy. 2020;21:21.
173. Abebe TB, Bhagavathula AS, Tefera YG, Ahmad A, Khan MU, Belachew SA, et al. Healthcare professionals' awareness, knowledge, attitudes, perceptions and beliefs about Ebola at Gondar University Hospital, Northwest Ethiopia: A cross-sectional study. J Public Health Afr. 2016;7(2).
174. Smith MW, Smith PW, Kratochvil CJ, Schwedhelm S. The Psychosocial Challenges of Caring for Patients with Ebola Virus Disease. Health security. 2017;15(1):104-9.
175. El Gaafary MM, Abd Elaziz KM, Abdel-Rahman AG, Allam MF. Concerns, perceived impacts and preparedness of health care workers in a referral hospital in Egypt in facing influenza (H1N1) epidemic. Journal of Preventive Medicine and Hygiene. 2010;51(3):105-9.
176. Austria-Corrales F, Cruz-Valdes B, Herrera-Kiengelher L, Garcia-Vazquez JC, Salas-Hernandez J. Burnout syndrome among Mexican physicians in training during a health emergency influenza virus A H1N1. Gaceta Medica de Mexico. 2011;147(2):97-103.
177. Tzeng HM, Yin CY. A crisis: Fear toward a possible H5n1 pandemic. Journal of Nursing Care Quality. 2008;23(2):177-83.
178. Wong TY, Koh GC, Cheong SK, Sundram M, Koh K, Chia SE, et al. A cross-sectional study of primary-care physicians in Singapore on their concerns and preparedness for an avian influenza outbreak. Annals of the Academy of Medicine, Singapore 37(6):458-64, 2008 Jun. 2008;37(6):458-64.
179. Tang L, Pan L, Yuan L, Zha L. Prevalence and related factors of post-traumatic stress disorder among medical staff members exposed to H7N9 patients. International Journal of Nursing Sciences. 2017;4(1):63-7.
180. Alsubaie S, Hani Temsah M, Al-Eyadhy AA, Gossady I, Hasan GM, Al-Rabiaah A, et al. Middle East Respiratory Syndrome Coronavirus epidemic impact on healthcare workers' risk perceptions, work and personal lives. Journal of infection in developing countries. 2019;13(10):920-6.
181. Poon E, Liu KS, Cheong DL, Lee CK, Yam LYC, Tang WN. Impact of severe acute respiratory syndrome on anxiety levels of frontline health care workers. Hong Kong Medical Journal. 2004;10(5):325-30.
182. Wong WCW, Lee A, Tsang KK, Wong SYS. How did general practitioners protect themselves, their family, and staff during the SARS epidemic in Hong Kong? Journal of Epidemiology and Community Health. 2004;58(3):180-5.

183. Wong TW, Yau JK, Chan CL, Kwong RS, Ho SM, Lau CC, et al. The psychological impact of severe acute respiratory syndrome outbreak on healthcare workers in emergency departments and how they cope. European journal of emergency medicine : official journal of the European Society for Emergency Medicine. 2005;12(1):13-8.
184. Chan AOM, Huak CY. Psychological impact of the 2003 severe acute respiratory syndrome outbreak on health care workers in a medium size regional general hospital in Singapore. Occupational Medicine. 2004;54(3):190-6.
185. Koh D, Lim MK, Chia SE, Ko SM, Qian F, Ng V, et al. Risk perception and impact of Severe Acute Respiratory Syndrome (SARS) on work and personal lives of healthcare workers in Singapore: what can we learn? Med Care. 2005;43(7):676-82.
186. Tan N, Goh L, Lee S. Family Physicians' Experiences, Behaviour, and Use of Personal Protection Equipment During the SARS Outbreak in Singapore: Do They Fit the Becker Health Belief Model? Asia-Pacific Journal of Public Health. 2006;18(3):49-56.
187. Tham KY, Tan YH, Loh OH, Tan WL, Ong MK, Tang HK. Psychological morbidity among emergency department doctors and nurses after the SARS outbreak. Hong Kong Journal of Emergency Medicine. 2005;12(4):215-23.
188. Verma S, Mythily S, Chan YH, Deslypere JP, Teo EK, Chong SA. Post-SARS psychological morbidity and stigma among general practitioners and traditional Chinese medicine practitioners in Singapore. Annals of the Academy of Medicine Singapore. 2004;33(6):743-8.
189. Lung F-W, Lu Y-C, Chang Y-Y, Shu B-C. Mental symptoms in different health professionals during the SARS attack: A follow-up study. Psychiatric Quarterly. 2009;80(2):107-16.
190. Wong WCW, Wong SYS, Lee A, Goggins WB. How to provide an effective primary health care in fighting against severe acute respiratory syndrome: The experiences of two cities. American Journal of Infection Control. 2007;35(1):50-5.
191. Grace SL, Hershenfield K, Robertson E, Stewart DE. The Occupational and Psychosocial Impact of SARS on Academic Physicians in Three Affected Hospitals. Psychosomatics: Journal of Consultation and Liaison Psychiatry. 2005;46(5):385-91.
192. Nickell LA, Crighton EJ, Tracy CS, Al-Enazy H, Bolaji Y, Hanjrah S, et al. Psychosocial effects of SARS on hospital staff: Survey of a large tertiary care institution. Cmaj. 2004;170(5):793-8.
193. Tolomiczenko GS, Kahan M, Ricci M, Strathern L, Jeney C, Patterson K, et al. SARS: coping with the impact at a community hospital. Journal of Advanced Nursing. 2005;50(1):101-10.

---

[1] PHQ (-2, -4, -9, -15) = Patient Health Questionnaire. A scale used to measure severity of somatic symptoms. Scores of 5, 10 and 15 represent cut off points for low, medium and high somatic symptom severity, respectively. PHQ-9 is a 9-item module for depressive symptoms. Total scores range from 0-27; a higher score is indicative of an increase in depressive severity (0-4: minimal. PHQ-2 is a brief version of this scale. PHQ-2 scores of ≥3 indicating possible cases. PHQ-4 is a brief version that measures anxiety and depression, with scores of ≥3 indicating a case.

[2] GAD (-2, -7) = Generalized Anxiety Disorder Scale. A scale for anxiety symptoms. Total scores range from 0-21; a higher score is indicative of an increase in anxiety symptoms; GAD-2 is a brief version of this scale. GAD-2scores range from 0-6 with a score ≥3 indicating possible cases.

[3] ISI = Insomnia Severity Index. A scale to assess sleeping problems. Total scores range from 0-28; a higher score is indicative of more severe sleep problems.

[4] PSS (-4,-10,-14) = Perceived Stress Scale. Three versions of PSS (PSS-4, PSS-10, PSS-14) comprise 4, 10 and 14 items. A global measure of stress constructed to measure the degree to which life events are judged as stressful. Higher scores indicate a higher degree of perceived stress.

[5] DASS-21 = Depression, Anxiety, and Stress Scale. Uses subscales for each of the three psychological domains. Each can be rated as normal, mild, moderate, and extremely severe.

[6] IES (-R) = Impact of Events Scale (Revised). The IES measures subjective signs of distress caused by traumatic events, including avoidance and intrusive characteristics on a sub-scale. Higher scores indicate higher psychological post-event morbidity.

[7] MSPSS = Multidimensional Scale of Perceived Support. A 12-item scale designed to measure perceived social support from three sources: family, friends and a significant other.

[8] SAS = Self-Assessed Anxiety Scale. Higher scores indicate increased levels of anxiety (normal, mild, moderate, severe).

[9] PTSD-SS = Post traumatic Stress Disorder Self-Rating. A score of 50 or higher indicates posttraumatic stress. Higher scores indicate more severe cases.

[10] Y-BOCS = Yale-Brown Obsessive-Compulsive Scale. A scale to measure obsessions and compulsions. Scores range from 0 (no symptoms) to 4 (severe symptoms). Total scores range from 0-40.

[11] SCL-90 (-R) = Symptom Checklist. Scale used to evaluate a range of psychological symptoms and distress.

[12] SDS = Self-rating Depression Scale. Higher scores indicate an increase in depressive symptoms.

[13] HADS = Hospital Anxiety and Depression Scale. A scale for anxiety and depression symptoms. Total scores range from 0 to 21; a higher score is indicative of an increase in anxiety and depression symptoms.

[14] VDAS = Van Dream Anxiety Scale. A scale for assessment of dream anxiety. Total scores range from 0 to 42; higher scores indicate more elevated dream anxiety level.

[15] PCL-5 = PTSD Checklist for DSM-5. A 20-item self-report scale to assess symptoms of PTSD.

[16] K (6, 10) = Kessler Psychological Distress Scale. A scale to measure psychological stress. Total scores range from 1-12 (minimal effect), 13-16 (intermediate effect) and 17-20 (huge effects). K-10 is the original scale, K-6 is a shorter version.

[17] Work-Family Conflict and Support Scale. A scale used to assess work-family conflict, social support and policy support.

[18] PSQI = Pittsburgh Sleep Quality Index. A scale used to assess sleep quality over a 1-mo interval. Total scores range from 0 – 21. A global score of 5 or more indicates poor sleep quality, the higher the score, the worse quality of sleep.

[19] CBI = Copenhagen Burnout Inventory. Measures burnout with three scales: personal burnout, work-related burnout and client-related burnout. CBI scores of 50-74 are moderate, 75-99 high and scores of 100 is considered severe burnout.

[20] BAI = Beck Anxiety Inventory. Measures anxiety levels. Total scores range from 0-63. Higher scores indicate higher levels of anxiety.

[21] SF (-36, -12) = Short Form Survey. Assess health-related quality of life, including mental and physical components. Total scores range from 0-100 with higher scores indicating higher quality of life. SF-12-Item is a shorter version of SF-36.

[22] AIS = Athens Insomnia Scale. A scale used to assess the severity of insomnia in the past month using diagnostic criteria from the International Classification of Diseases (ICD-10). Items are rated from 0 -3. Total scores ≥ 6 indicate the diagnosis of insomnia.

[23] STAI (-S, -T, -C) = State-Trait Anxiety Inventory measures anxiety related to events (state) and as a personal characteristic (trait). There are subscales from this measure, STAI-S (current state of anxiety), STAI-T (anxiety proneness), and STAIC (for children).

[24] PROMIS Scales = Patient-Reported Outcomes Measurement Information System. These scales are person-centered measures that evaluate and monitor physical, mental and social health in adults and children.

[25] CD-RISC = Connor-Davidson Resilience Scale – Chinese Version. A scale used to assess ability to respond and adapt to major life stresses. Total scores range from 0-100. Higher scores indicate higher resilience level.

[26] PRSF inventory = Inventory of Pandemic Related Stress Factors.

[27] CES-D (-R) = Center for Epidemiologic Studies Depression Scale. A Scale measuring symptoms of depression. Total scores range from 0 – 60, with higher scores indicating the presence of greater symptomatology.

[28] NASA-TLX = NASA Task Load Index. Multidimensional subjective tool to assess perceived workload.

[29] OFER-15 = Occupational Fatigue Exhaustion Recovery Scale. A scale used to measure work-related fatigue and distinguish between acute fatigue and chronic fatigue states. It has three subscales: chronic fatigue, acute fatigue, intershift recovery where scores range from 0 -100 with higher scores indicating more acute and chronic fatigue and greater intershift recovery.

[30] REQ = Recovery Experiences Questionnaire. Measures unwinding and recuperating from work during leisure time.

[31] SRQ-20 = Self Reporting Questionnaire. A 20-item self report screening tool developed by the World Health Organization to detect non-specific psychological distress over a 30-day period. It has a maximum total score of 20. A cut-off of 7/8 has been documented in multiple studies.

[32] SASRQ = Stanford Acute Stress Reaction Questionnaire. Evaluates anxiety and dissociation symptoms in the aftermath of traumatic event based on DSM-IV criteria for acute stress disorder. An algorithm allows calculation of diagnostic cut-off for acute stress disorder (ASD) based on DSM-IV criteria.

[33] MBI = Maslach Burnout Inventory – a psychological inventory pertaining to signs of occupational burnout. aMBI is an abbreviated form of the scale.

[34] PSM-25 = Psychological Stress Measure. A French questionnaire used to assess current stress levels. Total scores range from 25-200. High scores: ≥155 points, indicates maladaptation and the need for psychological correction; Average scores: 154–100 points; low scores < 100 points, indicates psychological adaptation to stresses).

[35] HWSS = Healthcare Workers Stigma Scale. A scale used to assess level of perceived stigma. Higher scores reflect higher level of perceived stigma.

[36] COPE/BRIEF-COPE = Coping Orientation to Problems Experienced. A self-report questionnaire designed to measure effective and ineffective ways to cope with stressful events. The BRIEF-COPE is a short version with 28 items of the original COPE which contains 60 items.

[37] GHQ = General Health Questionnaire. The GHQ is used to indicate psychological well-being and test for psychological morbidity. Original version of this questionnaire contains 60-items however there are abbreviated version with 30, 28, and 12 items also available.

[38] ProQOL = Professional Quality of Life Scale. 30-item self-report measure of positive and negative affects of helping others who experience suffering and trauma. The ProQOL has sub-scales for compassion satisfaction, burnout and compassion fatigue.

[39] MINI = Mini International Neuropsychiatric Interview. A short diagnostic structured interview developed in France and USA to explore 17 disorders under DSM-III-R diagnostic criteria.

[40] CGI (-S/-I) = Clinical Global Impressions Scale. An instrument to assess patient progress and treatment response over time. CGI-S refers to the Severity component which rates illness severity and the GCI-I refers to the improvement component which demonstrates change from baseline of treatment. Scores for the CGI-S are 1 = Normal, 2 = Borderline mentally ill, 3 = Mildly ill, 4 = Moderately ill, 5 – Markedly ill, 6 = Severely ill, 7 = Among most extremely ill. Scores for CGI-I are 1 = Very much improved, 2 = Much improved, 3 = Minimally improved, 4 = No change, 5 – Minimally worse, 6 = Much Worse, 7 = Very much worse.

[41] WHO-5 = World Health Organization Perceived Well-Being Index. Self-report scale that measures overall subjective well-being. Scores range from 0 (worst possible well-being) to 100 (best possible well-being).

[42] Healthcare Workers' Stress Related to Caring for Patients with a Highly Infectious Disease Scale. A scale with 4 subscales: worry and social isolation, discomfort from PPE, anxiety related to infection control and burden of caring for patients. Total scores range from 0-96 with higher scores indicating greater degree of stress; 46-96 = severe stress, 33-46 = moderate stress, 0-32 = low stress, 0 = no stress.

[43] BHS = Beck Hopelessness Scale. A 20-item self report inventory designed to measure hopelessness. Total scores can range from 0-20.

[44] WHOQOL-BREF = World Health Organization Quality of Life.

[45] PSI = Problem Solving Inventory. A 35-item instrument measuring perceptions regarding problem-solving abilities and problem-solving style. Total scores range from 32 to 192. Lower scores on each of the factors and on the total PSI score are considered more functional.

[46] BDI (-II) = Beck Depression Inventory. A 21-question multiple choice self-report inventory to measure the severity of depression

[47] FCV-19S = Fear of COVID-19 Scale. A 7-item scale used to assess fears of COVID-19 among individuals.

[48] Job Strain Scale Short Form. A 17-question scale composed of workload, control and social support dimensions. Scoring is between 1 (Never) and 5 (Always) in a 5-point Likert scale. The increase in total score corresponds to high job strain levels.

[49] WOC = Ways of Coping Questionnaire. A self-administered questionnaire consisting of 66 items grouped into 8 subscales

[50] EuroQol EQ-5D-3L. A standardized measure of health-related quality of life developed by the EuroQol group. The EQ-5D-3L has five dimensions, mobility, self-care, usual activities, pain/discomfort, and anxiety/depression.

[51] Jenkins Sleep Scale. Evaluates the frequency and intensity of sleep difficulties.

[52] Sleep_50 Questionnaire. Instrument designed to detect sleep disorders as listed in the DSM-IV.

[53] RS-14 = Resilience Scale. A 14-item resilience assessment with a 7-point Likert type scale. 1 (strongly disagree) to 7 (strongly agree).

[54] IU (-S) = Intolerance of Uncertainty Scale (S = Short Form). Measures uncertainty, ambiguous situations and the future. Items are rated on a 5-point Likert scale, 1 (not at all characteristic of me) to 5 (entirely characteristic of me).

[55] CISS = Coping Inventory for Stressful Situations. Measures engagement with various coping styles during stressful situations using a Likert scale ranging from 1 (Not at all) to 5 (Very much).

[56] CAS-1 = Cognitive Attentional Syndrome Scale-1. A 16-item scale to assess proportion of time in worry/rumination, threat monitoring, coping behaviours, levels of positive and negative metacognitive beliefs. A high CAS-1 score indicates an increased level of CAS activation.

[57] IIP = Inventory of Interpersonal Problems. A 127-item scale designed to determine sources of interpersonal stress.

[58] HAM-D = Hamilton Depression Rating Scale. Provides an indication of depression over time and effectiveness of antidepressants. Scoring is based on the 17-item scale, scores of 0-7 are normal, 8-16 suggest mild depression, 17-23 moderate depression and >24 severe depression.

[59] OLBI = Oldenburg Burnout Inventory. Scale used to measure the prevalence of burnout with two subscales, exhaustion and disengagement.

[60] Mini-Z = Mini-Z Burnout Assessment. A one-item Likert scale range to assess workplace burnout. A score of ≥ 3 is considered as burnout.

[61] Grit (-O/-S) = Grit-S is an 8-item short form of the original 12-item scale that measures perseverance – grit as an individual difference score. It has two subscales: consistency of interest and perseverance of effort.

[62] PFI = Stanford Professional Fulfilment Index. A 16-item survey that measures burnout work exhaustion and interpersonal disengagement and professional fulfillment

[63] NIOSH Quality of Worklife Questionnaire. Measures how work life and work experience with questions on workload, worker autonomy, layoffs, and job security, job satisfaction/stress and well-being.

[64] PWLS = Physician Work Life Study burnout item is a validated tool for self-rated burnout. (1 = "I enjoy my work"; 5 = "I am completely burned out").

[65] BRS = Brief Resilience Scale. A 6-item scale that measures the ability to bounce back from stress. Items are scored on a 5-point Likert scale. Average scores are low (1-2.99), normal (3-4.30), and high (4.31-5).

[66] WBI = Well-Being Index. A 9-item scale with 6 dimensions of distress and well-being. Scores range from -2 (lowest risk) to 9 (highest risk). Higher total scores reflect greater distress, lower meaning in work and lower satisfaction with work-life balance. Scores of ≥ 3 for physicians indicate increased burnout, depression, fatigue and lower quality of life.

[67] PC-PTSD = Primary Care Post Traumatic Stress Disorder Screen. A screening tool designed to identify persons with probable PTSD. Total scores range from 0-4; score ≥3 indicates a positive screen.

[68] Work-related Stress Test. A 12-item scale 6-point Likert scale derived from a range of tools that measure burnout syndrome. The questions are on somatizations. Higher individual and total scores correspond to worse evaluation, work related stress corresponds to a score of ≥ 25.

[69] AO Spine COVID-19 and Spine Surgeon Global Impact Survey. 73-item scale developed from a Delphi methodology with items on COVID-19 perceptions, institutional preparedness and response, personal and practice impact and future perceptions.

[70] TIPI = Ten-Item Personality Inventory. Assesses personality predisposition of participant (extraversion, agreeableness, conscientiousness, emotional stability and openness to experience).

[71] SWLS = Satisfaction with Life Scale. 5-item instrument measuring global cognitive judgements of satisfaction with one's life. Scores range from 5 (extremely dissatisfied with life) to 35 (extremely satisfied). A score of 20 represents a neutral point on the scale

[72] DAS = Death Anxiety Scale. Measures the extent of death anxiety. Scores range from 15 -35 are interpreted as low death anxiety, 36-55 moderate, and 56-75 high death anxiety.

[73] TSD PCL-C scores measure intrusive symptoms, avoidance symptoms, and hyper-arousal symptoms. High scores indicate high likelihood to show PTSD symptoms

[74] HSS = HIV Stigma Scale adapted for healthcare workers; 40-item measure of stigma and psychosocial aspects experienced by healthcare workers during the SARS outbreak based on four subscales: personalized stigma, disclosure concerns, negative self-image and concern with public attitudes towards healthcare workers.

[75] CHQ = Chinese Health Questionnaire; scores greater than or equal to 3 indicate mental illness symptoms.
