# Supplementary material for: Experiences and management of physician psychological symptoms during infectious disease outbreaks: a rapid review
Source: BMC Psychiatry. 2021 Feb 10;21:91. doi: 10.1186/s12888-021-03090-9 (PMC7875435; doi:10.1186/s12888-021-03090-9)
Supplement: Supplementary file 4 — Additional File 4 Quality analysis of included studies (n = 193). [file 12888_2021_3090_MOESM4_ESM.pdf]

Additional File 4. Quality analysis of included studies (n=193)

Cross-Sectional Studies								
**Newcastle-Ottawa Scale**								
	**Selection (Maximum 5 stars)**				**Comparability (Maximum 2 stars)**	**Outcome (Maximum 3 stars)**		**Quality Score**
**Author (Year)**	Representative of the sample	Sample size	Non-respondents	Ascertainment of the exposure (risk factor):	The subjects in different outcome groups are comparable, based on the study design or analysis. Confounding factors are controlled.	Assessment of outcome	Statistical test	Total stars (Max 10)
Abdessater (2020)	*	-	-	**	*	*	*	7
Abdulah and Mohammed (2020)	*	-	-	**	*	*	-	5
Abdulah and Musa (2020)	*	-	-	**	**	*	-	6
Abebe (2016)	*	-	-	*	*	*	*	5
Al Mahyijari (2020)	*	-	-	**	**	*	-	6
Al Sulais (2020)	*	-	-	**	*	*	*	7
Alahmadi (2020)	*	-	*	**	*	*	*	7
AlAteeq (2020)	-	*	-	**	**	*	*	7
Alhaj (2020)	*	-	-	**	**	*	-	6
Ali (2020)	*	-	-	**	*	*	-	5
Almater (2020)	*	-	-	**	**	*	*	7
Alnofaiey (2020)	*	-	-	**	*	*	-	5
Alsubaie (2019)	*	-	-	**	*	*	-	5
Amerio (2020)	*	-	-	**	*	*	*	7

Amin (2020)	*	*	-	**	*	*	*	7
An (2020)	-	-	-	-	*	*	*	3
Arafa (2020)	*	-	-	**	*	*	*	6
Arshad and Islam (2020)	*	-	-	**	*	*	*	6
Austria-Corrales (2011)	*	-	-	**	*	*	*	6
Awano (2020)	*	*	-	**	*	*	*	7
Ayub (2020)	-	-	-	*	*	*	-	3
Azoulay (2020)	*	-	-	**	*	*	-	5
Bahat (2020)	*	-	-	-	-	*	-	2
Bajaj and Solanki (2020)	*	-	-	**	-	*	-	4
Bakry (2020)	*	*	-	*	-	*	-	4
Banerjee (2020)	*	-	-	*	**	*	-	5
Barik (2020)	*	*	*	*	-	*	*	6
Bhargava (2020)	*	-	-	*	**	*	*	6
Bitonti (2020)	*	-	*	*	*	*	*	6
Bohlken (2020)	*	-	-	-	*	*	-	3
Büntzel (2020)	*	-	-	-	-	*	-	2
Buselli (2020)	-	-	-	**	**	*	*	6
Cai (2020)	*	-	-	**	*	*	-	6
Caliskan (2020)	*	*	-	**	**	*	-	7
Chan (2004)	*	-	-	**	*	*	*	6
Chang (2020)	*	*	*	*	**	*	*	8
Chatterjee (2020)	-	-	-	**	*	*	-	5
Chew (2020)	*	-	*	**	*	*	*	7
Civantos, A. (2020) USA	*	-	-	**	*	*	*	7

Civantos, A. (2020) Brazil	*	-	-	**	**	*	-	6
Collins (2020)	*	-	-	**	**	*	*	7
Cotrin (2020)	*	*	-	**	**	*	-	7
Coyle (2020)	*	-	-	-	*	*	-	3
Das (2020)	*	-	-	**	**	*	*	7
De Sio (2020)	*	*	-	**	**	*	*	8
Degraeve (2020)	*	-	-	**	*	*	*	6
Demirjian (2020)	*	-	-	**	**	*	*	7
Di Monte (2020)	*	-	-	**	**	*	-	6
Dimitriu (2020)	-	-	-	**	*	*	-	4
Diomidous (2020)	*	-	-	**	*	*	*	6
El Gaafary (2010)	*	-	-	**	*	*	-	5
Elbay (2020)	*	-	-	**	*	*	*	7
Elhadi and Msherghi (2020)	*	-	-	**	**	*	-	6
Elkholy (2020)	*	*	-	**	*	*	*	7
Enyama (2020)	*	*	*	*	*	*	-	6
Faderani (2020)	*	-	-	**	*	*	*	6
Fargen (2020)	*	-	-	*	**	*	*	6
Farooq (2020)	-	-	-	**	-	*	-	3
Fekih-Romdhane (2020)	*	-	-	**	*	*	*	6
Fitzpatrick (2020)	*	-	*	*	**	*	*	7
Florin (2020)	*	-	-	**	*	*	*	6
Foley (2020)	*	-	*	*	*	*	*	6
Gallopeni (2020)	-	-	-	**	*	*	-	4

Gangakhedkar and Solanki (2020)	*	*	-	**	*	*	-	6
Gill (2020)	*	-	-	-	-	*	-	2
Gokdemir (2020)	*	*	-	**	**	*	*	8
Grace (2005)	*	-	-	*	*	*	-	4
Guillen-Astete (2020)	*	-	-	**	**	*	*	7
Guiroy (2020)	*	-	-	**	*	*	-	5
Gupta (2020)	*	*	-	**	**	*	*	8
Hacimusalar (2020)	*	*	*	**	**	*	*	9
Halayam (2020)	*	-	-	**	*	*	-	5
Hasan (2020)	-	-	-	**	**	*	*	6
Hilmi (2020)	*	-	-	**	**	*	*	7
Holton (2020)	*	*	-	**	**	*	*	8
Huang, J. (2020)	*	-	-	**	*	*	-	5
Huffman (2020)	*	-	-	**	**	*	-	6
Imran (2020)	*	*	-	**	*	*	*	7
Jha (2020)	-	-	-	*	*	*	-	3
Jo (2020)	*	-	-	**	**	*	-	6
Johnson, A. (2020)	*	-	*	**	*	*	-	6
Johnson, S. (2020)	*	*	-	**	*	*	-	6
Juan (2020)	*	*	-	**	**	*	*	8
Kannampallil (2020)	*	-	-	**	**	*	*	7
Kapila (2020)	*	-	-	*	*	*	-	4
Khalafallah, A. (2020) Attendings	*	-	-	**	**	*	*	7

Khalafallah, A. (2020) Residents	-	-	-	**	*	*	-	4
Khanna (2020)	*	-	-	**	**	*	*	7
Khasne (2020)	-	-	-	**	**	*	*	6
Khattab (2020)	*	-	-	**	**	*	*	7
Khattab, M. and Abou-Madawi (2020)	-	-	-	*	*	*	-	3
Khusid (2020)	*	-	-	*	*	*	-	4
Koh (2005)	*	-	-	*	*	*	-	4
Korkmaz (2020)	-	-	-	**	*	*	-	4
Kramer (2020)	*	-	-	*	**	*	*	6
Kuo (2020)	-	-	-	**	**	*	-	5
Kurt (2020)	*	-	-	**	**	*	-	6
Lai (2020)	*	*	*	**	**	*	-	8
Lee (2020)	*	-	-	**	**	*	-	6
Li (2020)	*	-	-	**	**	*	*	7
Liu, Y. (2020)	*	-	-	**	**	*	-	6
Louie (2020)	*	-	-	**	**	*	-	6
Malgor (2020)	*	-	-	**	**	*	-	6
Martinez-Lopez (2020)	*	-	-	**	**	*	*	7
Milgrom, Y. (2020)	*	-	-	**	**	*	-	6
Milgrom, Y. and Richter, V. (2020)	*	-	*	**	**	*	*	8
Mishra (2020)	*	-	-	*	-	*	-	3
Mohd-Fauzi (2020)	*	*	-	**	**	*	*	8
Monterrosa-Castro (2020)	*	*	*	**	**	*	*	9
Mosheva (2020)	*	-	*	**	**	*	*	8

Naser (2020)	*	*	-	**	**	*	*	8
Naser Moghadasi (2020)	*	-	-	**	*	*	-	5
Nickell (2004)	*	-	-	**	**	*	-	6
Ning (2020)	*	-	-	**	**	*	*	7
Norton (2020)	*	-	-	*	**	*	*	6
O’Kelly (2020)	-	-	-	**	-	*	-	3
Odedra (2020)	*	-	-	*	-	*	-	3
Osama (2020)	-	-	-	**	*	*	-	4
Pandey (2020)	-	-	-	**	-	*	-	3
Payne (2020)	*	-	-	*	-	*	-	3
Pilar (2020)	*	-	-	**	*	*	-	5
Podder (2020)	*	-	-	**	*	*	-	5
Poon (2004)	*	-	-	**	*	*	-	5
Que (2020)	*	-	-	**	*	*	*	6
Rajan (2020)	*	-	-	*	*	*	-	4
Rajwa (2020)	*	-	-	*	**	*	-	5
Rimmer (2020)	-	-	-	-	*	*	-	2
Robbins (2020)	*	-	-	**	*	*	-	5
Rodriguez (2020)	*	-	*	**	*	*	*	7
Ruiz-Fernandez (2020)	*	-	-	**	**	*	-	6
Rymarowicz (2020)	*	-	-	*	**	*	*	6
Saadeh (2020)	-	-	-	*	**	*	*	5
Sahin (2020)	*	*	-	**	**	*	*	8
Sahu (2020)	*	-	-	**	*	*	-	5
Salopek-Ziha (2020)	-	-	-	**	**	*	-	5
Saracoglu (2020)	-	-	-	**	**	*	-	5

Sarma (2020)	*	-	-	**	-	*	-	4
Saurabh and Ranjan (2020)	*	-	-	*	-	*	-	3
Sayari (2020)	*	-	-	*	**	*	*	6
Schmulson (2020)	*	-	-	*	-	*	-	3
Schwartz (2020)	*	-	-	*	**	*	-	5
Shah, N. (2020)	*	-	-	**	**	*	-	6
Shah, S. (2020)	*	-	-	-	**	*	-	4
Shalhub (2020)	*	-	*	**	**	*	*	8
Shanghavi (2020)	-	-	-	**	-	*	-	3
Shao (2020)	*	-	-	**	*	*	*	6
Sharif (2020)	*	-	-	**	*	*	*	7
Sharma (2020)	*	-	*	**	**	*	*	8
Shechter (2020)	*	*	*	**	*	*	-	7
Sil (2020)	*	-	-	**	*	*	-	5
Singariya (2020)	*	*	*	**	**	*	*	9
Skoda (2020)	*	-	-	**	**	*	*	7
Song (2020)	*	-	-	**	**	*	-	6
Sorokin (2020)	*	-	-	**	*	*	-	5
Suryavanshi (2020)	*	-	-	**	**	*	*	7
Tan (2020)	-	-	*	**	**	*	-	6
Tang (2017)	*	-	-	**	*	*	-	5
Tas (2020)	*	-	-	**	*	*	-	5
Tham (2005)	*	-	-	**	*	*	-	5
Thomaier (2020)	*	-	-	**	**	*	-	6
Tolomiczenko (2005)	*	-	-	*	*	*	-	4
Tzeng (2008)	*	-	-	**	*	*	-	5

Urooj (2020)	-	-	-	**	-	*	-	3
Uvais (2020)	*	-	-	**	*	*	-	5
Uyaroglu (2020)	*	-	-	**	**	*	-	6
Vafaei (2020)	-	-	-	**	*	*	-	4
Vallee (2020)	*	*	*	**	**	*	*	9
Vanni (2020)	*	-	-	**	**	*	-	6
Verma (2004)	*	-	-	**	**	*	*	7
Wang, H. (2020)	*	*	-	**	**	*	*	8
Wang, Y. (2020)	*	-	-	**	**	*	*	7
Weiner (2020)	*	-	-	-	**	*	*	5
Wong (2008)	*	-	-	**	**	*	*	7
Wong (2004)	*	-	*	*	**	*	-	6
Wong (2005)	*	-	-	*	*	*	-	4
Wong (2007)	-	-	-	*	**	*	*	5
Wu (2020)	-	-	-	-	-	*	-	1
Yang (2020)	*	-	-	**	**	*	*	7
Zerbini (2020)	-	-	-	**	*	*	-	4
Zhang (2020)	*	-	-	**	**	*	*	7
Zhu (2020)	*	-	-	**	*	*	-	5

Cohort Studies									
	Newcastle-Ottawa Scale								
	Selection (Maximum 5 stars)				Comparability (Maximum 2 stars)	Outcome (Maximum 3 stars)			Quality Score
Author (Year)	Representatives of exposed cohort	Selection of non - exposed cohort	Ascertainment of exposure	Demonstration that the outcome of interest was not present at start of study	Comparability of cohorts on the basis of the design or analysis	Assessment of outcome	Was follow-up long enough for outcomes to occur?	Adequacy of follow-up cohorts	Total stars (Max 10)
de Wit (2020	*	*	-	-	**	-	*	*	6
Kelker (2020)	*	*	*	*	*	-	*	-	6
Li (2020)	*	*	-	*	*	*	*	-	6
Lung (2009)	-	-	-	-	*	*	*	*	4
Zhao (2020)	*	*	-	*	*	-	*	*	6

Qualitative Studies										
	JBI Critical Appraisal Checklist for Qualitative Research									
**Author (Year)**	Is there congruity between stated philosophical perspective & research methodology?	Is there congruity between research methodology & research question / objectives?	Is there congruity between research methodology & methods used to collect data?	Is there congruity between research methodology and representation & analysis of data?	Is there congruity between research methodology & interpretation of results?	Is there a statement locating the researcher culturally or theoretically?	Is the influence of the researcher on the research, and vice-versa, addressed?	Are participants, and their voices, adequately represented ?	Is the research ethical according to current criteria? Is there evidence of ethical approval by an appropriate body?	Do the conclusions drawn in the research report flow from the analysis or interpretation of the data?	**Overall appraisal**
Khot (2020)	Unclear	Yes	Unclear	Unclear	Unclear	No	No	Unclear	No	Yes	Include
Liu (2020)	Yes	Yes	Yes	Yes	Yes	No	No	Yes	Yes	Yes	Include
Rashid (2020)	Yes	Yes	Yes	Yes	Yes	No	No	Yes	Yes	Yes	Include
Smith (2017)	Unclear	Yes	Yes	Yes	Yes	No	No	Yes	Yes	Yes	Include
Tan (2006)	Unclear	Yes	Yes	Yes	Yes	No	No	Yes	No	Yes	Include
Xu (2020)	Yes	Yes	Yes	Yes	Yes	Yes	No	No	Yes	Yes	Include

Pre- and Post-Test Studies										
	JBI Critical Appraisal Checklist for Pre-test, Post-test Design									
**Author (Year)**	Is it clear in the study what is the 'cause' and what is the 'effect' (i.e. there is no confusion about which variable comes first)?	Were the participants included in any comparisons similar?	Were the participants included in any comparisons receiving similar treatment/care, other than the exposure or intervention of interest?	Was there a control group?	Were there multiple measurements of the outcome both pre- and post-test intervention/exposure?	Was follow up complete and if not, were differences between groups in terms of their follow up adequately described and analyzed?	Were the outcomes of participants included in any comparisons measured in the same way?	Were outcomes measured in a reliable way?	Was appropriate statistical analysis used?	Overall appraisal
Liu, Z. (2020)	Yes	Yes	Yes	No	No	Yes	Yes	Yes	Yes	Include
